# Supplementary material for: Clinical management of community-acquired meningitis in adults in the UK and Ireland in 2017: a retrospective cohort study on behalf of the National Infection Trainees Collaborative for Audit and Research (NITCAR)
Source: BMJ Open. 2022 Jul 12;12(7):e062698. doi: 10.1136/bmjopen-2022-062698 (PMC9315913; doi:10.1136/bmjopen-2022-062698)
Supplement: Supplementary data [file bmjopen-2022-062698supp001.pdf]

Site of Data collection	Names and Grades (at time of data collection) of contributors
Birmingham Heartlands Hospital, University Hospitals Birmingham NHS Foundation Trust	Amy Chue, SpR<br>Ed Moran, Consultant<br>Karishma Gokani, CMT
North Manchester General Hospital	Joseph Thompson, SpR<br>Katherine Ajdukiewicz, Consultant
Oxford University Hospitals	Victoria Ward, SpR<br>Lucinda Barrett, Consultant
Cheltenham General Hospital	Frances Edwards, CMT<br>Adam Usher, Consultant
Royal Alexandra Hospital, Paisley	Mairi McLeod, Consultant<br>Ramandeep Singh, medical student<br>Su su Htwe, SpR
Leicester Royal Infirmary, Leicester	Benedict Rogers, SpR<br>Grace Duane, Medical Student<br>Martin Wiselka, Consultant<br>Nicholas Wong, SpR
NHS Lothian	Elen Vink, SpR<br>Jennifer Poyner, SpR<br>Jenni Crane, Consultant<br>Ollie Lloyd, SpR<br>Emma Chisholm, SpR
Countess of Chester Hospital	Ildiko Kustos, Consultant<br>Ruth McEwen, Consultant<br>Sam Sutton, CMT
University Hospitals Plymouth Trust	Lewis Jones, Consultant<br>Robert Tilley, Consultant
Addenbrookes hospital, Cambridge University Hospitals NHS Foundation Trust	M. Estee Torok, Honorary Consultant<br>Isobel Ramsay, SpR
Hull University Teaching Hospitals NHS Trust	Monica Ivan, Consultant<br>Joshua York<br>Jennifer Ansett<br>Maithili Varadarajan<br>Celestine Eshiwe, SpR
London King's College	Amanda Fife, Consultant<br>Stephanie Harris, SpR<br>Ryan Jayesinghe, medical student<br>Priya Sekhon
Aintree University Hospital, Liverpool	James Cruise, SpR<br>Susan Larkin, Consultant
Worcestershire Royal Hospital	Shivani Kanabar, Medical student<br>Ernest Mutengesa, Medical Student<br>Mirella Ling, Consultant<br>Christopher Green, Consultant
Bristol Royal Infirmary, University Hospitals Bristol NHS Foundation Trust	Martin Williams, Consultant<br>Matthew Stevens, CMT

Victoria hospital, Kirkcaldy	David Griffith, Consultant<br>Naomi Bulteel, SpR
Northumbria Healthcare NHS Foundation Trust	Charlotte Milne, SpR<br>Jayanta Sarma, Consultant
Ninewells hospital, Dundee	Aline Wilson, SpR<br>John Shone, Consultant<br>Lynn Urquhart, Consultant<br>Sahar Eldirdiri, SpR
Royal Preston Hospital, Lancashire Teaching Hospitals NHS Foundation Trust	Alison Muir, Consultant<br>Leila White, Clinical Scientist
Sheffield teaching Hospitals	Jody Aberdein, Consultant<br>Phillip Simpson, SpR
Shrewsbury and Telford Hospital NHS Trust	Hnin Hay Mar<br>John Bowen<br>Keying Tan<br>Eint Shwe Zin thein<br>Mahmoud Aziz
University Hospital North Midlands	Anthony Cadwgan, Consultant<br>Brendan Davies, Consultant<br>Daniel White, SpR<br>Natasha Weston, SpR<br>Salman Zeb, CMT
St George's Hospital, London	Angela Houston, Consultant<br>Imogen Fordham, clinical fellow<br>Terry John Evans, SpR<br>Louise Wootton, Physician's associate
Nottingham University Hospitals NHS Trust	David Turner, Consultant<br>Iona Willingham, SpR
Birmingham Queen Elizabeth Hospital	Aimee Johnson, SpR<br>Nimal Wickramasinghe, Consultant
Salford Royal Infirmary, Salford	Ashley Horsley, SpR<br>Eamonn Trainor, Consultant<br>Olivier Gaillemin, Consultant
University Hospital Southampton NHS Foundation Trust	Andrew Rosser, Consultant<br>Nicholas J Norton, SpR
Royal Blackburn Hospital, East Lancashire Hospitals NHS Trust	Iain Crossingham, Consultant<br>Katie Cheung, Medical Student<br>Megan Duxbury, CMT
Queen Elizabeth University Hospital, NHS Greater Glasgow and Clyde	Ashutosh Deshpande, Consultant<br>Emilie Bellhouse, FY2<br>Kamaljit Khalsa, SpR
Imperial College School of Medicine and Imperial college Healthcare NHS trust	Helena Brezovjakova, Medical Student<br>Emma McLean, medical student<br>Tanmay, Kanitkar, CMT<br>Nicholas Davies, Consultant<br>Alexsander Dawidziuk, Medical Student

St James University hospital, Leeds	Eloisa Mclaughlin, Medical student
	Joanna Allen, Consultant
	Razan Saman, SpR
	Sarah Kelly, SpR
Royal Liverpool University Hospital, Liverpool	Hugh Adler, SpR
	Sylviane Defres, Consultant
Arrowe Park Hospital, Wirral	David Harvey, Consultant
	Elshadai Ejere, FY2
Queen's hospital, Romford	Aarti Shah, Consultant
	Yiwen Soo, FY1
Raigmore Hospital, Inverness	Wendy Beadles, Consultant
	Heather Sturgeon, Medical student
	Brodie Cameron, Medical Student
James Cook University Hospital, Middlesbrough	Ben Tomlinson, SpR
	David Chadwick, Consultant
University Hospital Monklands	Claire McGoldrick, Consultant
	Katie McDowell, FY2
Cumberland infirmary, Carlisle	Alastair Miller, Consultant
	Clive Graham, Consultant
	Mpho Molosiwa, FY2
Newcastle Upon Tyne NHS Foundation Trust	Ewan Hunter, Consultant
	Ruth Owen, Medical Student
	Katherine Flack
Airedale hospital, Airedale	Adrian Kennedy, Consultant
Bradford Royal Infirmary, Bradford	Amy Robinson, Consultant
	Phoebe Cross, SpR
	Fay Perry
University Hospital Wales	Vithusha Inpadhas
Aberdeen Royal Infirmary	Ali Khan, SpR
	Sarathy Selvam, FY2
	Vhairi Bateman, Consultant
	Jeremy Wong, Medica Student
Lancaster Royal Infirmary	Henry Wu, FY2
	Monika Pasztor, Consultant
Whittington Hospital, London	Trupti Patel, Consultant
	Ajanthiha Karunakaran, Medical Student
Russells Hall Hospital, Dudley	Basma Soliman, CT1
	Hassan Paraiso, Consultant
Glasgow Royal Infirmary	Mairi McLeod, Consultant
	Su su Htwe, SpR
	Anna Smith
James Paget University Hospitals NHS Foundation Trust	Andrew Blanshard, CMT
	Harish Reddy, Consultant
Portsmouth Hospitals University NHS Trust	Avneet Shahi, SpR
	Helen Chesterfield, Consultant
	Oliver Bannister, CMT

Withybush hospital, Haverford West	Ben Schroeder, Medical Student<br>Ken Woodhouse, Consultant
Ashford and St Peter's NHS Foundation Trust	Jan Coebergh, Consultant<br>Viva Levee, FY2
Mater Misericordiae University Hospital, Dublin	Eavan Muldoon, Consultant<br>Rhea O'regan, SPR<br>Tee Keat Teoh, SpR
Newham Hospital, Barts Health NHS Trust	Sathyavani Subbarao, SpR<br>Simon Tiberi, Consultant<br>Caryn Rosmarin
London UCL and Hospital for Tropical diseases at University College London Hospitals NHS Foundation Trust.	Jayne Ellis, SpR<br>Lucy Bell, CMT<br>Robert Heyderman, Consutant
Barts Health NHS Trust	Jonathan Lambourne, Consultant<br>Emma McGuire, SpR<br>Robert Serafino, Consultant
Guy's and St Thomas' NHS Foundation Trust	Anna Goodman, Consultant<br>Ishaan Bhide, FY1<br>Karanjeet Sagoo, Medical Student
Whipps Cross, Barts Health NHS Trust	Mark Melzer, Consultant<br>Maria Krutikov, SpR
The Royal Free Hospital, London	Indran Balakrishnan, Consultant<br>Susan Hopkins, Consultant<br>Tim Jones, SpR
Trafford General Hospital, Manchester University NHS Foundation Trust	Kajal Patel, Medical Student<br>Barzo Faris, Consultant
William Harvey Hospital, East Kent	Graeme Calv er, Consultant<br>Ricky Singh, Medical Student<br>Hazel Sanghvi, Medical Student
Tameside General Hospital	Mohamed Eltayeb, Clinical Fellow<br>Rathur Haris, Consultant
