# Supplementary material for: Clinical management of community-acquired meningitis in adults in the UK and Ireland in 2017: a retrospective cohort study on behalf of the National Infection Trainees Collaborative for Audit and Research (NITCAR)
Source: BMJ Open. 2022 Jul 12;12(7):e062698. doi: 10.1136/bmjopen-2022-062698 (PMC9315913; doi:10.1136/bmjopen-2022-062698)
Supplement: Supplementary data [file bmjopen-2022-062698supp002.pdf]

Supplementary table 1 . Risk factors for Listeria stratified by aetiology.

	Total cohort N (%)	Bacterial meningitis N (%)	Viral meningitis N (%)	Other meningitis† N (%)	P value
**N**	1,471 (100)	302 (21)	615 (42)	553 (38)	-
**Age >60 years**	235 (16)	126 (42)	27 (4)	79 (14)	<0.001
**Number immunocompromised by disease/medication***	60 (4)	14 (5)	18 (3)	28 (5)	0.23
**Number with Diabetes mellitus**	64 (4)	30 (10)	11 (2)	22 (4)	<0.001
**Number with a history of alcohol excess**	36 (2)	21 (7)	3 (0.5)	12 (2)	<0.001

†= other meningitis category included all patients without a confirmed bacterial or viral pathogen

*=Conditions listed as immunocompromising conditions included haematological malignancy (n=8), Other malignancy (n=8), solid organ transplant (n=6), liver cirrhosis (n=1), HIV (n=9), Pregnancy (n=2). Medicaion listed included Steroids (n=7), tocilizumab, ecolizumab and infliximab (n=6), Methotrexate (n=8), Mycophenolate (n=2), Azathioprine (n=3), 'chemotherapy' (n=4). (some patients had more than one immunocompromising condition/medication).

Supplementary table 2: Multivariate analysis of the association between baseline co-variates and in-hospital mortality in 266 patients with bacterial meningitis confirmed by CSF analysis using logistic regression modelling:

Baseline co-variate	N	In-hospital mortality N (%)[1]	Crude OR for in-hospital mortality (95% CI)	P-value	Adjusted OR for in-hospital mortality (95% CI)*	P-value†
**Sex**						
Male	147	15 (10.2)	1			
Female	118	7 (5.93)	0.55 (0.22-1.42)	0.21		
**Age group**						
≤ 18 years	16	0 (0)	-			
19 – 59 years	136	10 (7.35)	1			
≥ 60 years	113	12 (10.6)	1.50 (0.62-3.61)	0.37		
**Blood culture positive**						
No	130	8 (6.15)	1		1	
Yes	135	14 (10.4)	1.76 (0.71-4.38)	0.21	1.46 (0.58-3.71)	0.42
**GCS ≤ 13[2]**						
No	106	3 (2.83)	1		1	
Yes	132	17 (12.9)	5.05 (1.41-18.2)	0.006	4.41 (1.24-15.7)	0.009
**IV dexamethasone given[3]**						
No	124	10 (8.06)	1		1	
Yes	137	11 (8.03)	0.99 (0.41-2.43)	0.99	1.02 (0.41-2.52)	0.96
**IV dexamethasone given if *Strep.pneumoniae*[4]**						
No	62	8 (12.9)	1		1	
Yes	89	8 (8.99)	0.67 (0.23-1.89)	0.44	0.68 (0.24-1.94)	0.48
**Final diagnosis *S. pneumoniae***						
No	107	5 (4.46)	1		1	
Yes	136	17 (11.1)	2.67 (0.95-7.55)	0.05	2.37 (0.84-6.67)	0.08
**ITU admission[5]**						
No	129	4 (3.01)	1		1	
Yes	113	18 (13.7)	5.14 (1.65-16.0)	0.002	4.44 (1.44-13.6)	0.003

*adjusted for sex and age group
† P-value from LRT comparing models with and without primary exposure variable
1 = One participant had missing outcome data
2 = 28/266 (10%) participants did not have a GCS recorded
3 = 4/266 (1%) participants had missing data on IV dexamethasone administration
4 = 2/154 (1%) participants with confirmed S. pneumoniae meningitis had missing data on IV dexamethasone administration
5 = 2/266 (0.7%) participants had missing data on ITU admission

Supplementary table 3: Multivariate analysis of the association between baseline co-variates and in-hospital mortality in 359 patients with bacterial meningitis using the Spanos criteria^ using logistic regression modelling:

Baseline co-variate	N	In-hospital mortality N (%)[1]	Crude OR for in-hospital mortality (95% CI)	P-value	Adjusted OR for in-hospital mortality (95% CI)*	P-value†
**Sex**						
Male	199	28 (14.1)	1			
Female	159	13 (8.18)	0.54 (0.27-1.09)	0.08		
**Age group**						
≤ 18 years	21	0 (0)	-			
19 – 59 years	192	18 (9.38)	1			
≥ 60 years	145	23 (15.9)	1.82 (0.94-3.52)	0.07		
**Blood culture positive**						
No	188	14 (7.45)	1		1	
Yes	170	27 (15.9)	2.35 (1.18-4.68)	0.01	1.93 (0.96-3.89)	0.06
**GCS ≤ 13[2]**						
No	163	9 (5.52)	1		1	
Yes	156	28 (17.9)	3.74 (1.67-8.36)	<0.001	3.19 (1.44-7.09)	0.003
**IV dexamethasone given[3]**						
No	189	26 (13.8)	1		1	
Yes	162	14 (8.64)	0.59 (0.30-1.18)	0.13	0.57 (0.28-1.14)	0.11
**IV dexamethasone given if *Strep.pneumoniae*[4]**						
No	73	16 (21.9)	1		1	
Yes	96	11 (11.5)	0.46 (0.19-1.08)	0.07	0.47 (0.20-1.10)	0.08
**Final diagnosis *S. pneumoniae***						
No	187	13 (6.95)	1		1	
Yes	171	28 (16.4)	2.62 (1.30-5.29)	0.005	2.29 (1.14-4.63)	0.02
**ITU admission[5]**						
No	192	9 (4.69)	1		1	
Yes	163	32 (19.6)	4.97 (2.24-11.0)	<0.001	4.43 (2.03-9.68)	<0.001

*adjusted for sex and age group
† P-value from LRT comparing models with and without primary exposure variable
1 = One participant had missing outcome data
2 = 40/359 (11%) participants did not have a GCS recorded
3 = 7/359 (2%) participants had missing data on IV dexamethasone administration
4 = 2/172 (1%) participants with confirmed S. pneumoniae meningitis had missing data on IV dexamethasone administration
5 = 4/359 (1%) participants had missing data on ITU admission
^ - Spanos criteria use various parameters to allow patients who have not had an aetiological agent to be assumed to be likely bacterial in nature.
