# Supplementary material for: Clinical management of community-acquired meningitis in adults in the UK and Ireland in 2017: a retrospective cohort study on behalf of the National Infection Trainees Collaborative for Audit and Research (NITCAR)
Source: BMJ Open. 2022 Jul 12;12(7):e062698. doi: 10.1136/bmjopen-2022-062698 (PMC9315913; doi:10.1136/bmjopen-2022-062698)
Supplement: Author's manuscript [file bmjopen-2022-062698.draft_revisions.pdf]

BMJ Open is committed to open peer review. As part of this commitment we make the peer review history of every article we publish publicly available.

When an article is published we post the peer reviewers' comments and the authors' responses online. We also post the versions of the paper that were used during peer review. These are the versions that the peer review comments apply to.

The versions of the paper that follow are the versions that were submitted during the peer review process. They are not the versions of record or the final published versions. They should not be cited or distributed as the published version of this manuscript.

BMJ Open is an open access journal and the full, final, typeset and author-corrected version of record of the manuscript is available on our site with no access controls, subscription charges or pay-per-view fees (http://bmjopen.bmj.com).

If you have any questions on BMJ Open's open peer review process please email info.bmjopen@bmj.com

BMJ Open

Clinical management of community-acquired meningitis in adults in the United Kingdom and Ireland in 2017: a retrospective cohort study on behalf of the National Infection Trainees Collaborative for Audit and Research (NITCAR).

Journal:	*BMJ Open*
Manuscript ID	bmjopen-2022-062698
Article Type:	Original research
Date Submitted by the Author:	21-Mar-2022
Complete List of Authors:	Ellis, Jayne; UCL,<br>Harvey, David; Wirral University Teaching Hospital NHS Foundation Trust<br>Defres, Sylviane; University of Liverpool; Royal Liverpool and Broadgreen Hospitals NHS Trust<br>Chandna, Arjun ; Cambodia Oxford Medical Research Unit, Angkor Hospital for Children, ; University of Oxford Centre for Tropical Medicine and Global Health,<br>MacLachlan, Eloisa; University of Leeds; National Student Association of Medical Research<br>Solomon, Tom; University of Liverpool, Neurological Science, Medical Microbiology<br>Heyderman, Robert; University College London, Division of Infection and Immunity; University of Malawi College of Medicine, Malawi Liverpool Wellcome Trust Clinical Research Programme<br>McGill, Fiona; University of Liverpool, Institute of Infection and Global Health; Leeds Teaching Hospitals NHS Trust, Infectious Diseases study group, NAMM; British Infection Association, Collaborative authorship group
Keywords:	Diagnostic microbiology < INFECTIOUS DISEASES, Molecular diagnostics < INFECTIOUS DISEASES, Infectious disease/HIV < NEUROLOGY, INTERNAL MEDICINE, Protocols & guidelines < HEALTH SERVICES ADMINISTRATION & MANAGEMENT

SCHOLARONE™
Manuscripts

For peer review only - http://bmjopen.bmj.com/site/about/guidelines.xhtml

BMJ

I, the Submitting Author has the right to grant and does grant on behalf of all authors of the Work (as defined in the below author licence), an exclusive licence and/or a non-exclusive licence for contributions from authors who are: i) UK Crown employees; ii) where BMJ has agreed a CC-BY licence shall apply, and/or iii) in accordance with the terms applicable for US Federal Government officers or employees acting as part of their official duties; on a worldwide, perpetual, irrevocable, royalty-free basis to BMJ Publishing Group Ltd ("BMJ") its licensees and where the relevant Journal is co-owned by BMJ to the co-owners of the Journal, to publish the Work in this journal and any other BMJ products and to exploit all rights, as set out in our licence.

The Submitting Author accepts and understands that any supply made under these terms is made by BMJ to the Submitting Author unless you are acting as an employee on behalf of your employer or a postgraduate student of an affiliated institution which is paying any applicable article publishing charge ("APC") for Open Access articles. Where the Submitting Author wishes to make the Work available on an Open Access basis (and intends to pay the relevant APC), the terms of reuse of such Open Access shall be governed by a Creative Commons licence – details of these licences and which Creative Commons licence will apply to this Work are set out in our licence referred to above.

Other than as permitted in any relevant BMJ Author's Self Archiving Policies, I confirm this Work has not been accepted for publication elsewhere, is not being considered for publication elsewhere and does not duplicate material already published. I confirm all authors consent to publication of this Work and authorise the granting of this licence.

For peer review only

Clinical management of community-acquired meningitis in adults in the United Kingdom and Ireland in 2017: a retrospective cohort study on behalf of the National Infection Trainees Collaborative for Audit and Research (NITCAR).

Jayne Ellis, Specialty trainee[1], David Harvey, Consultant[2], Sylviane Defres, Consultant[3,4,10], Arjun Chandna, Specialty trainee[5,6], Eloisa Maclachlan, Medical Student[7,8], Tom Solomon, Professor[4,9,10], Robert S Heyderman (0000-0003-4573-449X), Professor[1,11], Fiona McGill (0000-0002-0903-9046), Consultant*[4,10,12] on behalf of the NAMM (national audit of meningitis management) group.
*Corresponding author: f.mcgill@nhs.net

1. Hospital for Tropical Diseases, University College London Hospitals NHS Foundation Trust, London, UK.
2. Wirral University Teaching Hospital NHS Foundation Trust, UK
3. Liverpool University Hospitals NHS Foundation Trust, Liverpool, UK
4. Institute of Infection, Veterinary and Ecological sciences, University of Liverpool, UK

Nuffield department of Medicine, University of Oxford

5. Department of Clinical research, London School of Hygiene and Tropical Medicine, London, UK
6. Mahidol-Oxford Tropical Medicine Research Unit, Mahidol University, Bangkok, Thailand
7. University of Leeds, Leeds, UK
8. National Student Association for Medical Research
9. The Walton Centre NHS Foundation Trust, Liverpool, UK
10. NIHR Health Protection Research Unit for Emerging and Zoonotic infections, University of Liverpool, Liverpool, UK
11. Research Department of Infection, Division of Infection and Immunity, University College London, London, UK.
12. Department of Infection and Travel Medicine and Department of Microbiology, Leeds Teaching Hospitals NHS Trust, Leeds, UK

*Corresponding author: f.mcgill@nhs.net.

Corresponding author: Dr Fiona McGill, Leeds Teaching Hospitals NHS Trust, Beckett Street, Leeds, LS9 7TF

f.mcgill@nhs.net.

COI: None to declare

Key words: meningitis, adults, bacteria, antibiotics, management, guidelines

Word Count: 2901

For peer review only

Abstract

Objectives: To assess current practice of suspected community acquired bacterial meningitis in adults in the UK.

Design: Retrospective cohort

Setting: 64 UK and Irish hospitals

Participants: 1,471 adults with community acquired meningitis of any aetiology, admitted in 2017.

Results: None of the audit standards, from the 2016 UK Joint Specialists Societies guideline on the diagnosis and management of meningitis, were met in all cases. With respect to 20 of the 30 assessed standards, the clinical management provided for patients was in line with recommendations in less than 50% of cases. 45% of patients had blood cultures taken within an hour of admission, 0.5% had a lumbar puncture within one hour, 26% within 8 hours. 28% had bacterial molecular diagnostic tests on CSF. Median time to first dose of antibiotics was 3.2 hours (IQR 1.3-9.2). 82% received empirical parenteral cephalosporins. 55% ≥ 60 years and 31% of immunocompromised patients received anti-*Listeria* antibiotics. 21% of patients received steroids. Of the 1,471 patients, 21% had confirmed bacterial meningitis. Amongst those with bacterial meningitis, pneumococcal aetiology, admission to intensive care and initial Glasgow Coma Scale score less than 14 were associated with in-hospital mortality (aOR 2.08, 95% CI 0.96 – 4.48; aOR 4.28, 95% CI 1.81 – 10.1; aOR 2.90, 95% CI 1.26 – 6.71 respectively). Dexamethasone therapy was weakly associated with a reduction in mortality in both those with proven bacterial meningitis (aOR 0.57, 95% CI 0.28 – 1.17) and with pneumococcal meningitis (aOR 0.47, 95% CI 0.20 – 1.10).

Conclusion: This large study demonstrates that clinical care for patients with meningitis in the UK is not in line with current evidence-based national guidelines. Diagnostics and therapeutics should be targeted for quality improvement strategies. Additionally, work should be done to

improve the reach and impact of guidelines, once published, to ensure they translate into changes in practice.

For peer review only

Strengths and Limitations of this study

- This is the largest national study of the management of meningitis in the UK published to date
- The study includes all suspected community acquired bacterial meningitis, allowing assessment of early clinical care prior to an aetiological diagnosis being made
- The study is widely translatable, and therefore representative of practice within the UK and Ireland
- The study is limited by its retrospective design which brings associated recall bias and some missing data
- The study may also be limited by the self-selection of the sites included

Introduction

Acute bacterial meningitis is a medical emergency associated with considerable death and disability in the UK(1). Successful immunisation programmes targeting *Haemophilus influenzae* type b, *Streptococcus pneumoniae* and *Neisseria meningitidis* means that community acquired bacterial meningitis, particularly in children and adolescents, is now relatively rare (2). The incidence of bacterial meningitis in adults in England is estimated to be approximately 1-1.25 per 100,000 population overall, exceeding 9 per 100,000 in people over 70 years (2, 3).

Early recognition of meningitis, appropriate investigation, and treatment, saves lives (4, 5). It is therefore essential that front-line clinicians, who may not encounter meningitis very often, are vigilant and have a high index of suspicion to minimise poor outcomes. To help frontline medical staff who are seeing patients with suspected meningitis, the UK guidelines on the diagnosis and management of acute meningitis and meningococcal sepsis in immunocompetent adults were published in 2016(6). The guidelines provide readily accessible, comprehensive, evidenced-based recommendations. Previously published data suggest that clinical care delivered in the UK is frequently non-adherent to guidelines(7, 8). A recent UK study of viral meningitis also highlighted a large amount of inappropriate brain imaging prior to lumbar punctures (LPs) and long delays in performing the LP (3, 9). Inadequate use of

For peer review only

molecular diagnostics and HIV-testing have also been highlighted as areas for improvement(3). The increasing risk of multi-drug resistant bacteria, an ageing population susceptible to a wider variety of bacteria (e.g. *Listeria monocytogenes, Escherichia coli* and *Klebsiella pneumoniae*)(2) and the greater appreciation that viruses are a common cause of meningitis in UK adults(10), make diagnostics essential. Reports from outside the UK have shown improvements in outcomes following guideline publication and implementation (11). We carried out a retrospective observational study with the dual aims of i) assessing current clinical practice regarding diagnosis and management of adult patients with suspected community acquired bacterial meningitis, and ii) to identify areas for improvement.

Methods

Hospitals in the UK were invited to take part in this study via the National Infection Trainees Collaborative for Audit and Research (NITCAR) network, the UK Meningitis study network, the British Infection Association (BIA) and through personal contacts. Eligible patients were identified via hospital coding data, laboratory data, or a combination of both. Data from patients aged 16 or over who presented with suspected acute community acquired bacterial meningitis during 2017 were eligible for screening. Patients who met our case definition for confirmed acute meningitis, regardless of aetiology, were eligible for inclusion (box 1). Definitions are as previously published(3). Many interventions are performed prior to knowing the final diagnosis, therefore, we included all types of meningitis in the analysis, including viral and those in whom no pathogen was identified. This allowed us to assess the entire clinical pathway of patients presenting with possible bacterial meningitis, although some would be ultimately diagnosed with a different aetiology, or no confirmed aetiology at all.

For peer review only

Box 1: Inclusion and exclusion criteria for cases of meningitis.

A meningitis case was defined as:

(1) Patients with a cerebrospinal fluid (CSF) WCC >4 x10^6 cells/L (regardless of whether a pathogen was identified or not) and a clinical suspicion of meningitis at the time OR

(2) in the case of bacterial meningitis, symptoms and signs of meningitis with a significant pathogen in the CSF (culture or PCR) or blood regardless of CSF leukocyte count.

Patients with the following diagnoses were excluded:

(1) Cryptococcal meningitis;

(2) Tuberculous meningitis;

(3) Nosocomial meningitis (defined as meningitis that occurs during a hospital admission or within 30 days of discharge or meningitis associated with indwelling devices in the central nervous system)

(4) Encephalitis (defined as altered consciousness for >24 with no other cause found and two or more of the following signs: fever or history of fever (≥38°C) during the current illness; seizures or focal neurological signs (with evidence of brain parenchyma involvement); CSF pleocytosis (>4 × 10^6 cells per L); EEG suggesting encephalitis; and neuroimaging suggestive of encephalitis).

Standards indicative of good practice were taken from the 2016 UK Joint Specialists Societies guideline on the diagnosis and management of meningitis and meningococcal sepsis in immunocompetent adults, and the Standards in Microbiological Investigations on the processing of cerebrospinal fluid (B27) (6, 12). For each standard, the number of patients as a proportion of the total cohort who received clinical care in line with the standard is reported. A second adjusted analysis taking account of missing data is also reported, whereby the number of patients as a proportion of the cohort with available data who received clinical care in line with the standard was reported.

For peer review only - http://bmjopen.bmj.com/site/about/guidelines.xhtml

Data were collected using electronic case report forms on REDcap™, a password protected central web-based database system. All microbiological diagnostic procedures were performed at the local hospital laboratory for each participating site using locally approved procedures. All data were anonymised and recorded under a unique participant identification number.

Ethics approval: As all data were anonymised individual patient consent and ethical approval was not required. The study was registered with each site's clinical governance department in line with local procedure.

Statistical analyses: Descriptive statistics were used to summarize data. Categorical data were summarized using counts and percentages. Denominators presented are based on available data, where incomplete case records were submitted by contributing sites. For continuous variables, means and ranges or medians and inter-quartile ranges (IQRs) are presented depending on the distribution of the data. Categorical data were analysed using Chi squared or Fisher's exact test. Continuous data were analysed using t-tests, Mann Whitney U or Kruskall Wallis depending on the distribution of the data. Regression analysis was used to identify potential risk factors associated with poor outcomes.

Patient and Public Involvement. Although there was no direct involvement of patients and public in this study the Meningitis Research Foundation, a key advocacy group for patients are represented in the authorship of the original guidelines and will be key in the dissemination of the results and the subsequent call to improve practice. Preliminary results have been shared with the Meningitis Research Foundation and some of their members.

Results

1,471 patients from 64 hospitals throughout the UK and Ireland took part. The hospitals ranged in size from small district generals to larger teaching hospitals. The mean number of beds was 846 (range 230-2000). The hospitals who took part in England comprised 45% of the

For peer review only

total acute bed base in England, (42,612/94,827(13)). Females accounted for 57% and the median age was 34 years (IQR 26,49). Confirmed viral meningitis occurred in 615 (42%) and 303 had confirmed bacterial meningitis (21%). More than one third of patients (n=553) fulfilled the case definition (box 1) but had no confirmed microbiological diagnosis and were therefore categorised as meningitis of unknown aetiology. *Streptococcus pneumoniae* and *Neisseria meningitidis,* were the most common bacterial pathogens, where a cause was found, accounting for 172 (57%) and 76 (25%) of cases respectively. *Haemophilus influenzae* (serotypes unknown) was found in 14 cases. *Enteroviruses* were the most common viral pathogens occurring in 429 (69%) of all confirmed viral meningitis. *Herpes simplex virus-2* was the second most common viral pathogen detected in 97 (16%) of viral cases. Baseline demographics and clinical characteristics are shown in table 1.

For peer review only

Table 1: Baseline demographics, timing of key investigations and clinical outcomes of 1,471 adults presenting with suspected meningitis:

	Total cohort N (%)	*Bacterial meningitis N (%)*	*Viral meningitis N (%)*	*Other† N (%)*	*P value[1]*
**N**	1,471 (100)	303 (21)	615 (42)	553 (38)	-
**Median age (IQR)**	34 (26 – 49)	54 (36 – 65)	31 (25 – 37)	34 (26 – 48)	< 0.001
**Male**	625 (43)	173 (57)	214 (35)	238 (43)	< 0.001
**In patient mortality**	48 (3)	38 (13)	2 (0.3)	8 (1.4)	< 0.001
**ITU admission**	192 (13)	157 (53)	4 (0.7)	31 (6)	< 0.001
Median Admission GCS (IQR)	15 (14-15)	13 (9 – 15)	15 (15-15)	15 (15-15)	< 0.001
**Median time (hours) from admission to first antibiotics (IQR)**	2.7 (0.9 – 8.3)	1.5 (0.4 – 5.3)	3.2 (1.3 – 8.3)	3.3. (1 – 12.5)	< 0.001
**Median time (hours) from admission to blood cultures (IQR)**	1 (0.3 – 4)	0.7 (0.2 – 2.4)	1 (0.3 – 3.7)	1.4 (0.3 – 6.1)	0.003
**CT head prior to LP**	1,094 (94)	207 (93)	459 (94)	428 (95)	0.55
**Median time (hours) from admission to LP (IQR)**	16.4 (7.9 – 26.7)	14.8 (7.7 – 29.8)	14.3 (7.5 – 22.6)	20 (8.8 – 35.8)	< 0.001
**Adjunctive dexamethasone**	300 (21)	150 (50)	69 (11)	81 (15)	< 0.001
Median CSF leucocyte count (IQR)	140 (44-399)	930 (235.5 – 3062.5)	122 (48 – 276)	85 (26.8 – 250.3)	< 0.001
Median CSF protein (IQR)	0.68 (0.46 - 1.21)	3.25 (1.4 – 5.8)	0.63 (0.45-0.9)	0.6 (0.4-1.0)	< 0.001
Median CSF glucose (IQR)	3.2 (2.8-3.7)	2.1 (0.95 – 3.45)	3.2 (2.9 – 3.6)	3.3 (3.0-3.8)	< 0.001

†= other meningitis category included all patients without a confirmed bacterial or viral pathogen
1 = for continuous variables, the Kruskal-Wallis test was used to compare medians across groups, and for categorical variables Chi squared tests were used.

Adherence to specific standards of good practice is shown in table 2. None were adhered to 100% of the time. Two thirds of the standards (n=20) had less than or equal to 50% adherence.

For peer review only

Table 2. Adherence to audit standards^

**Immediate management**				
	**Number achieved standard/total number of patients analysed**	**% of total**	**Number achieved standard/total number of patients evaluable***	**% of number evaluable**
1. The patient's conscious level should be documented using the Glasgow coma scale.	1,283/1,471	87%	1283/1448	89%
2. Blood cultures should be taken as soon as possible and within 1 h of arrival at hospital	326/1,471[1]	22%	326/767[2]	42%
3. LP should be performed within 1 h of arrival at hospital provided that it is safe to do so	8/1,471[3]	0.5%	8/1379[4]	0.6%
4. Antibiotic treatment should be commenced within the first hour	207/1471[5]	14%	207/1083[6]	19%
5. Patients with meningitis and meningococcal sepsis should be cared for with the input of an infection specialist such as a microbiologist or a physician with training in infectious diseases and/or microbiology	1,148/1,471[7]	78%	1148/1464	78%
**Investigations**				
6. Blood culture should be sent	977/1,471	66%	977/1469	67%
7. Blood Pneumococcal PCR should be sent	211/1,471	14%	211/1460	14%
8. Blood Meningococcal PCR should be sent	232/1,471	16%	232/1461	16%
9. CSF opening pressure should be documented	655/1,428[8]	46%	655/1361[9]	48%
10. CSF glucose with concurrent plasma glucose should be sent	607/1,428[8]	43%	607/1415	43%
11. CSF protein should be sent	1,358/1,428[8]	95%	1358/1420	96%
12. Microscopy of the CSF should take place within 2 hours of the lumbar puncture	596/1428[8]	42%	596/1203[10]	50%
13. CSF for pneumococcal PCR should be sent in all cases of suspected bacterial meningitis	412/1,428[8]	29%	412/1418	29%
14. CSF for Meningococcal PCR should be sent in all cases of suspected bacterial meningitis	434/1,428[8]	30%	434/1418	31%
15. A swab of the posterior nasopharyngeal wall should be obtained as soon as possible, and sent for meningococcal culture, in all cases of suspected meningococcal meningitis/sepsis	54/1,471	4%	54/1463[11]	4%
16. All patients with meningitis should have an HIV test	646/1,471	44%	646/1459[12]	44%
**Treatment**				
17. All patients with suspected meningitis or meningococcal sepsis should be given ceftriaxone or cefotaxime	1039/1471[13]	71%	1039/1423[14]	73%
18. If the patient has, within the last 6 months, been to a country where penicillin resistant pneumococci are prevalent, IV vancomycin 15-20 mg/kg should be added 12-hourly (or 600 mg rifampicin 12-hourly IV or orally) [15]	See footnote			

19. Those aged 60 or over should receive 2 g IV ampicillin/amoxicillin 4-hourly in addition to a cephalosporin [1B].	55/233	24%	55/197[16]	28%
20. Immunocompromised patients (including diabetics and those with a history of alcohol misuse) should receive 2 g IV ampicillin/amoxicillin 4-hourly in addition to a cephalosporin.	26/115[17]	23%	26/99[18]	26%
21. If there is a clear history of anaphylaxis to penicillins or cephalosporins give IV chloramphenicol 25 mg/kg 6- hourly	14/37	38%	14/30[19]	47%
22. If Streptococcus pneumoniae is identified continue with IV benzylpenicillin 2.4 g 4 hourly, 2 g ceftriaxone IV 12 hourly or 2 g cefotaxime IV 6-hourly	114/172	66%	114/145[20]	79%
23. If N. meningitidis is identified 2 g ceftriaxone IV 12 hourly, 2 g cefotaxime IV 6-hourly or 2.4 benzylpenicillin IV 4-hourly may be given as an alternative	52/76	68%	52/68[21]	76%
24. If the patient is not treated with ceftriaxone (in meningococcal disease), a single dose of 500 mg ciprofloxacin orally should also be given	0/2	0%	0/2	0%
25. If *Listeria monocytogenes* is identified Give 2 g ampicillin/amoxicillin IV 4-hourly and continue for at least 21 days. Co-trimoxazole 10-20 mg/kg or chloramphenicol are alternatives in cases of anaphylaxis to beta lactams.	4/7	57%	4/6	67%[22]
26. If *Haemophilus influenzae* is identified continue 2g ceftriaxone IV 12-hourly or 2 g cefotaxime IV 6-hourly for 10 days	9/14	64%	9/13	69%[23]
27. 10 mg dexamethasone IV 6 hourly should be started on admission, either shortly before or simultaneously with antibiotics.	67/1,471	5%	67/1435[24]	5%
28. If pneumococcal meningitis is confirmed dexamethasone should be continued for 4 days.	34/172[25]	20%	34/158[26]	22%
**Critical Care**				
29. The following patients should be transferred to critical care - those with a rapidly evolving rash, those with a GCS of 12 or less and those with uncontrolled seizures	151/203[27]	74%	151/203	74%
**Notification**				
30. All cases of meningitis (regardless of aetiology) should be notified to the relevant public health authority	236/1,471	16%	236/1465	16%

Notes.
^Only those audit standards that could be measured from the data collected. *excludes those where there was missing data and/or where not relevant. 1. Only 977 patients had blood cultures taken. 2. Excluding those who did not have blood cultures taken and where data was missing. 3. 1428 patients had a LP. 4. Excludes those who did not have an LP and where data was not available. 5. 82 patients had data consistent with having antibiotics prior to admission, this might be due to confusion about whether admission meant admission to the emergency department or admission to a ward, or it may represent data entry error therefore, these figures are not included. 6. 388 patients did not receive any antibiotics at all. 7. 310 (21%) of patients were admitted under an infection specialist, all others received consulting advice only. 8. 43 people did not have an LP. 9. Missing data on 67. 10. 43 had no LP, 97 missing data, 128 time of microscopy was before or at the same time as the LP. 11. Performed in 15/76 (20%) of proven meningococcal cases. 12. 9 known HIV positive and 3 missing data. 13. 285 patients were not given any antibiotics at all. 14. 48 patients who were definitely given antibiotics had missing data on which antibiotics they were given. 15. Using mainland Europe data only and with reference to ECDC data – 101 patients were documented to have travelled to a mainland European country within the previous 6 months. Travel history was not documented at all in 822 cases (56%). Of the 101 patients who had travelled to mainland Europe 54 (54%) had been to a country with a rate of penicillin resistant pneumococci of >5% (2017 data). 5/52 had no antibiotics. 0/47 had antibiotics to cover for penicillin resistant pneumococci. 16. 233 patients were aged over 60 but only 207 received antibiotics. Missing data for 10, 108 received amoxicillin at some point but only 55 received the correct dose. 17. Not including those >=60. 18. 15 did not received any antibiotics and missing data on 1. 19. 7 patients had no antibiotics at all. 20. 27 patients had insufficient antibiotic data. 21. 8 patient had insufficient antibiotic data. 22. 1 patient had insufficient antibiotic data. 23. Insufficient antibiotic data on 1 person. 24. Missing data on 36 – 11 on whether dexamethasone was received or not, 21 on the dose given and 4 on the timing. 25. Only 18 were given the correct dose (10mg). Some received dexamethasone for longer than 4 days. 26. Missing data on 14 individuals. 27. 7/11 patient with progressing rash, 131/176 patients with GCS <13 and 13/16 patients with uncontrolled seizures.

For peer review only

Overall in-hospital mortality was 3% and was considerably higher in patients with bacterial meningitis (13%), and pneumococcal meningitis in particular (16%). Mortality in viral meningitis was 0.3% and 1.45% in those with meningitis of unknown aetiology**.** Just over half (157) of those with confirmed bacterial meningitis required admission to an intensive care unit (ICU).

Use of diagnostics

A few patients, 43, did not have an LP, of whom 26 (60%) had no contraindication (as specified in the 2016 joint specialties guidelines and shown in box 2). Almost all these patients, who did not have an LP, had either a positive blood culture (36, 84%) or a positive blood PCR test (5, 12%).

Box 2: Indications for neuroimaging before lumbar puncture (LP) in suspected meningitis.

(1) Focal neurological signs
(2) Presence of papilloedema
(3) Continuous or uncontrolled seizures
(4) GCS ≤ 12

Contra-indications for immediate LP were uncommon and occurred in 299 (20%) patients. Glasgow coma score (GCS) ≤ 12 was the most common contra-indication for immediate LP reported in 143 (10%), followed by focal neurological signs in 38 (3%). A further 70 (7%) had other indications to delay LP. Neuroimaging prior to LP happened in 1094 of 1158 patients (94%), 911 (83%) of whom had no guideline-specified indication. Neuroimaging was performed a median of 11 hours post arrival at hospital (IQR 4-21). Median time from admission to LP was 16.5 hours (IQR 8 – 27). Only 6 patients had an LP within 1 hour of arrival at hospital and only 326 (26%) within 8 hours.

For peer review only

Median time from LP to CSF microscopy was 2 hours (IQR 1.1-3.2). Time from LP to CSF analysis was significantly quicker when performed at on-site laboratories when compared to centralised laboratory processing (median 1.65 hours (IQR 1.0 - 2.8) compared to 2.95 hours (IQR 2.0 - 3.8) $p < 0.001$).

Fewer than one third of patients had pneumococcal (412, 28%) and meningococcal polymerase chain reaction (PCR) (434, 29.5%) performed on their CSF. Pneumococcal PCR was done on blood in 211 (14%) patients, and meningococcal PCR in 232 (16%). 646 patients (44%) patients had a documented HIV test. Four of these were positive – two of whom had pneumococcal meningitis, one of whom had enteroviral meningitis and one had meningitis of unknown aetiology. Nine patients were previously known to be HIV positive.

Blood cultures were taken from 66% (n=977) of patients with 45% (n=438) having them taken within one hour of arrival at hospital.

Treatment

285 patients (19%) did not receive antibiotics, most of whom had either viral meningitis (163) or lymphocytic meningitis with no aetiology identified (105). The remaining 1,186 patients received at least one dose of antibiotics. The median time from hospital admission to first dose of antibiotics was 3.2 hours (IQR 1.3,9.2). Amongst the patients who received antibiotics the antimicrobials were commenced within an hour of arrival at hospital for approximately one fifth of patients (207/1000). In confirmed bacterial meningitis cases, 92 patients (36%) received antibiotics within an hour of arrival.

Adherence with guideline specified empirical antibiotic regimens was good with 912 (80%) receiving a third-generation cephalosporin. Data is missing on antibiotic type for 47 patients. Of the 197 patients aged 60 years and over who received antibiotics, 108 (55%) received ampicillin or amoxicillin; only 55 (28%) of those had the correct dose and dosing frequency as recommended for *Listeria monocytogenes* meningitis. Similarly, only 36 (31%)

For peer review only

223 of the immunocompromised patients, who were aged under 60, (n=115) received any
224 ampicillin or amoxicillin for anti-*Listeria* cover. The supplementary table shows details
225 regarding risk factors for Listeria.

226 Only 300 patients (20%) received adjunctive steroids as recommended. Steroids were
227 given more frequently in patient with confirmed bacterial meningitis in 150 (50%) cases. In
228 patients with pneumococcal meningitis 97 patients (57%) received steroids.

229 *Clinical outcomes*

230 On multivariate analysis, having a confirmed diagnosis of bacterial meningitis was
231 strongly associated with in-hospital mortality. Adjusting for age and sex, confirmed bacterial
232 meningitis was associated with 26 times the odds of in-hospital mortality compared to those
233 with other forms of meningitis (aOR 25.9, 95% CI 5.93 – 113.0), including those with no
234 aetiology identified.

235 In patients with confirmed bacterial meningitis, on univariate analyses, in-hospital
236 mortality was associated with a positive blood culture (cOR 2.21, 95% CI 1.04 – 4.67); GCS ≤
237 13 (cOR 3.24, 95% CI 1.39 – 7.52), confirmed *Streptococcus pneumoniae* meningitis (cOR
238 2.37, 95% CI 1.10 – 5.11); and ICU admission (cOR 4.81, 95% CI 1.99 – 11.60). These
239 associations remained despite multivariate adjustment for age and sex (table 3).

240

For peer review only

Table 3: Multivariate analysis of the association between baseline co-variates and in-hospital mortality in 303 patients with confirmed bacterial meningitis using logistic regression modelling:

Baseline co-variate	N	In-hospital mortality N (%)[1]	Crude OR for in-hospital mortality (95% CI)	P-value	Adjusted OR for in-hospital mortality (95% CI)*	P-value†
**Sex**						
Male	173	26 (15.1)	1			
Female	130	12 (9.23)	0.57 (0.27-1.18)	0.13		
**Age group**						
≤ 18 years	18	0 (0)				
19 – 59 years	159	18 (11.3)	1			
≥ 60 years	126	20 (16.0)	1.49 (0.75 – 2.96)	0.25		
**Blood culture positive**						
No	137	11 (8.09)	1		1	
Yes	166	27 (16.3)	2.21 (1.04 – 4.67)	0.03	1.87 (0.87 – 4.01)	0.10
**GCS ≤ 13[2]**						
No	124	8 (6.45)	1		1	
Yes	148	27 (18.2)	3.24 (1.39 – 7.52)	0.004	2.90 (1.26 – 6.71)	0.008
**IV dexamethasone given[3]**						
No	149	23 (15.4)	1		1	
Yes	150	14 (9.40)	0.57 (0.27 – 1.16)	0.11	0.57 (0.28 – 1.17)	0.12
**IV dexamethasone given if *Strep.pneumoniae*[4]**						
No	73	16 (21.9)	1		1	
Yes	97	11 (11.5)	0.46 (0.20 – 1.08)	0.07	0.47 (0.20 – 1.10)	0.08
**Final diagnosis *S. pneumoniae***						
No	131	10 (7.63)	1		1	
Yes	172	28 (16.4)	2.37 (1.10 – 5.11)	0.02	2.08 (0.96 – 4.48)	0.05
**ITU admission[5]**						
No	144	7 (4.86)	1		1	
Yes	157	31 (19.7)	4.81 (1.99 – 11.60)	< 0.001	4.28 (1.81 – 10.1)	< 0.001

*adjusted for sex and age group
† P-value from LRT comparing models with and without primary exposure variable
1 = One participant had missing outcome data
2 = 31/303 (10%) participants did not have a GCS recorded
3 = 4/303 (1%) participants had missing data on IV dexamethasone administration
4 = 2/172 (1%) participants with confirmed S. pneumoniae meningitis had missing data on IV dexamethasone administration
5 = 1/303 (0.3%) participants had missing data on ITU admission

On both univariate and multivariate analyses (adjusted for age and sex), in patients with confirmed bacterial meningitis, the administration of dexamethasone was associated with a reduction in in-hospital mortality (aOR 0.57, 95% CI 0.28 – 1.17, p 0.12). When this analysis was restricted to include only those with confirmed *Streptococcus pneumoniae* meningitis, those who received dexamethasone had a reduced odds of in-hospital mortality (aOR 0.47, 95% CI 0.20 – 1.10, p 0.08). Neither association reached statistical significance.

Discussion

This large national study evaluated the clinical management of adults with community acquired meningitis throughout the UK and Ireland. Current practice falls short of the recommendations in the 2016 UK guidelines(6). This is a concern for all patients but is of a particular worry in bacterial meningitis. The management of bacterial meningitis is time critical(4, 14). Delays in receiving antibiotics and having an LP, the unnecessary use of brain imaging, a lack of appropriate antibiotics in those at risk of *Listeria* and the low rate of steroid administration are areas for significant improvement.

Most patients in this study were given antibiotics prior to LP. Even taking this into consideration, the median door to antibiotic time was still over three hours. The optimal timing of antibiotics in bacterial meningitis is not known precisely but we do know that delays lead to increased mortality (4, 5, 14). A delay of over three hours has previously been associated with a 14-fold increase risk of death(14).

Delays in obtaining CSF are associated with a reduction in pathogen detection, increased exposure to unnecessary anti-infectives, prolonged hospital stays and increased mortality (4, 6, 15). In most cases brain imaging is not indicated in adults with suspected community-acquired meningitis (4) however, in our cohort, a significant number of patients had unnecessary scans. Although complications following LP are rare(16, 17), there may be an unfounded fear of cerebral herniation following LP, even in those with no clinical features of brain shift, which is leading to this excessive use of imaging(18). Education programs, along with quality improvement measures, are essential to reduce the potentially harmful overuse of neurological imaging. In addition, it is essential that we optimise meningitis care pathways to ensure that clinicians have the time, space and equipment required to performed LPs in a timely and safe manner (3, 19).

For peer review only

CSF culture positivity rates decline substantially when LP is delayed (3, 15). PCR can detect bacterial DNA in CSF for several days after antibiotics have been administered. In the UK 50% of meningococcal disease is diagnosed on PCR alone(20). Therefore, it is alarming that PCR was used, in our cohort, as a diagnostic modality in so few patients. Meningitis specific investigation order-sets using electronic ordering, and/or reflex laboratory testing to increase use of molecular diagnostics should be considered to reduce opportunities for missed microbiological diagnoses. There is the potential for increased use of rapid technologies that can be used on site with minimal technical skill required(21). Having a rapid tests on site has been shown to reduce bed days with significant cost savings in enteroviral meningitis (22). Further research evaluating rapid diagnostic tests in other types of meningitis with clinically relevant outcomes is needed. We also need to increase the offer of HIV testing in patients with meningitis, as less than half the patients had a documented HIV test. Incident HIV diagnoses were made in our cohort amongst patients presenting with bacterial, viral, and unknown cause meningitis.

Corticosteroids have been shown to reduce mortality in pneumococcal meningitis (23). We saw a 50% reduction in mortality in patients with pneumococcal meningitis who were given steroids, albeit not reaching statistical significance. It is of concern that well-evidenced, well-established therapies known to improve outcome, including mortality, are only being given to just over half of those who might benefit. A protocolised, goal-directed therapy bundle, including the use of corticosteroids and appropriate antibiotics warrants evaluation in the UK.

Although this is a large multi-national study, there are some limitations. NHS trusts self-selected themselves for inclusion and we cannot rule out any significant differences with trusts that did not. However, given that 64 hospitals were involved and good representation from throughout the nations of the UK (and Ireland) we don't think this selection bias limits the generalisability of our findings. We used well-established, published case definitions of

For peer review only

meningitis to minimise information bias, however misclassification of cases remains possible especially in the cases without a confirmed microbiological diagnosis. Our case definitions allowed us to include anyone suspected of having meningitis (of any cause) as objectively it is often difficult to differentiate between viral and bacterial meningitis at the point of initial assessment. However, it is possible that there may have been differences in presentation between those with confirmed bacterial meningitis, those with confirmed viral meningitis and those with no confirmed aetiology that meant they were managed in different ways. This study was not powered to look at the differences between all the different aetiologies. Finally, because this was a retrospective study, our analysis may have been subject to errors resulting from recall bias and missing data. A prospective national study would have been challenging to execute and it is likely that there would have been ascertainment bias in time and geography. We therefore believe that, due the large sample size along with the use of electronic hospital coding and laboratory data to ascertain cases, the risk of recall bias is low, and our retrospective data is representative of practice within the UK.

In conclusion this is, to our knowledge, the largest UK study of adult patients with meningitis. Awareness of practice guidelines for relatively rare acute medical conditions such as meningitis is low and this study has demonstrated that despite clear, freely accessible guidelines, clinical care is not in line with evidence-based recommendations. There is considerable room for improvement. Whilst we recognise that guidelines do not improve practice on their own, we do recommend that the findings from this study are strongly considered in the development of the new National Institute for Clinical Excellence (NICE) guideline on meningitis currently being developed, which for the first time, will include adults as well as children. Given the widespread adoption of NICE endorsed guidelines and quality standards to improve the quality of clinical practice, we anticipate that a NICE guideline will improve awareness and uptake of good practice in the short term. In addition to education,

For peer review only

which has limited impact on changing behaviour, UK hospitals should use quality improvement methods to improve management of patients with suspected meningitis. We suggest a national strategic improvement plan should focus on the following key areas: timely use of diagnostics; appropriate antibiotics in at risk populations and the use of adjunctive steroids. The integrated use of electronic systems to standardize optimal use of diagnostics, and management bundles may offer additional opportunities to improve outcomes. Each site that has been involved in this study has been asked to implement site specific changes and re-evaluate for any improvements in practice.

For peer review only

Author contributions

Ellis J: Methodology, Data collection and curation, formal analysis, Investigation, Writing – Original Draft Preparation. Harvey D: Methodology including pilot data, data collection, reviewing and approving final draft. Defres S: Methodology including development of original audit tool and guidelines, data collection, reviewing and approving final draft. Chandna A: Methodology, reviewing and approving final draft. Maclachlan E: Methodology, data collection, reviewing and approving final draft. Solomon T: Methodology including development of original guidelines and audit tool, reviewing and approving final draft. Heyderman RS: Conceptualization, Methodology, Supervision, Writing – Review & Editing. McGill F: Conceptualization, Methodology, Data collection and curation, Investigation, formal analysis, Writing –Original Draft Preparation. Responsible for overall content as guarantor.

The corresponding author attests that all listed authors meet authorship criteria and that no others meeting the criteria have been omitted.

The Corresponding Author has the right to grant on behalf of all authors and does grant on behalf of all authors, a worldwide licence to the Publishers and its licensees in perpetuity, in all forms, formats and media (whether known now or created in the future), to i) publish, reproduce, distribute, display and store the Contribution, ii) translate the Contribution into other languages, create adaptations, reprints, include within collections and create summaries, extracts and/or, abstracts of the Contribution, iii) create any other derivative work(s) based on the Contribution, iv) to exploit all subsidiary rights in the Contribution, v) the inclusion of electronic links from the Contribution to third party material where-ever it may be located; and, vi) licence any third party to do any or all of the above.

Competing interests: All authors have completed the ICMJE uniform disclosure form at www.icmje.org/coi_disclosure.pdf and declare: no support from any organisation for the submitted work; no financial relationships with any organisations that might have an interest in the submitted work in the previous three years; no other relationships or activities that could appear to have influenced the submitted work.

The lead author and corresponding author affirm that the manuscript is an honest, accurate, and transparent account of the study; no important aspects of the study have been omitted.

Original data can be shared on request.

For peer review only

Acknowledgements

TS is supported by the National Institute for Health Research (NIHR) Health Protection Research Unit in Emerging and Zoonotic Infections (Grant No. NIHR200907), NIHR Global Health Research Group on Brain Infections (No. 17/63/110), and the UK Medical Research Council's Global Effort on COVID-19 Programme (MR/V033441/1)

Funding

This research received no specific grant from any funding agency in the public, commercial or not-for-profit sector.

Data Availability Statement

Data can be made available to other researchers on reasonable request to the authors.

For peer review only

379 List of contributors in NAMM

380

Site of Data collection	Names and Grades (at time of data collection) of contributors	Number of patients' data contributed
Birmingham Heartlands Hospital, University Hospitals Birmingham NHS Foundation Trust	Amy Chue, SpR	60
	Ed Moran, Consultant	
	Karishma Gokani, CMT	
North Manchester General Hospital	Joseph Thompson, SpR	54
North Manchester General Hospital	Katherine Ajdukiewicz, Consultant	54
Oxford University Hospitals	Victoria Ward, SpR	50
Oxford University Hospitals	Lucinda Barrett, Consultant	50
Cheltenham General Hospital	Frances Edwards, CMT	47
Cheltenham General Hospital	Adam Usher, Consultant	47
Royal Alexandra Hospital, Paisley	Mairi McLeod, Consultant	45
Royal Alexandra Hospital, Paisley	Ramandeep Singh, medical student	45
Royal Alexandra Hospital, Paisley	Su su Htwe, SpR	45
Leicester Royal Infirmary, Leicester	Benedict Rogers, SpR	42
Leicester Royal Infirmary, Leicester	Grace Duane, Medical Student	42
Leicester Royal Infirmary, Leicester	Martin Wiselka, Consultant	42
Leicester Royal Infirmary, Leicester	Nicholas Wong, SpR	42
NHS Lothian	Elen Vink, SpR	42
NHS Lothian	Jennifer Poyner, SpR	42
NHS Lothian	Jenni Crane, Consultant	42
NHS Lothian	Ollie Lloyd, SpR	42
NHS Lothian	Emma Chisholm, SpR	42
Countess of Chester Hospital	Ildiko Kustos, Consultant	40
Countess of Chester Hospital	Ruth McEwen, Consultant	40
Countess of Chester Hospital	Sam Sutton, CMT	40
University Hospitals Plymouth Trust	Lewis Jones, Consultant	38
University Hospitals Plymouth Trust	Robert Tilley, Consultant	38
Addenbrookes hospital, Cambridge University Hospitals NHS Foundation Trust	M. Estee Torok, Honorary Consultant	37
Addenbrookes hospital, Cambridge University Hospitals NHS Foundation Trust	Isobel Ramsay, SpR	37
Hull University Teaching Hospitals NHS Trust	Monica Ivan, Consultant	36
Hull University Teaching Hospitals NHS Trust	Joshua York	36
Hull University Teaching Hospitals NHS Trust	Jennifer Ansett	36
Hull University Teaching Hospitals NHS Trust	Maithili Varadarajan	36
Hull University Teaching Hospitals NHS Trust	Celestine Eshiwe, SpR	36
London King's College	Amanda Fife, Consultant	36
London King's College	Stephanie Harris, SpR	36
London King's College	Ryan Jayesinghe, medical student	36
London King's College	Priya Sekhon	36
Aintree University Hospital, Liverpool	James Cruise, SpR	35
Aintree University Hospital, Liverpool	Susan Larkin, Consultant	35
Worcestershire Royal Hospital	Shivani Kanabar, Medical student	35
Worcestershire Royal Hospital	Ernest Mutengesa, Medical Student	35
Worcestershire Royal Hospital	Mirella Ling, Consultant	35

For peer review only

Worcestershire Royal Hospital	Christopher Green, Consultant	35
Bristol Royal Infirmary, University Hospitals Bristol NHS Foundation Trust	Martin Williams, Consultant	33
Bristol Royal Infirmary, University Hospitals Bristol NHS Foundation Trust	Matthew Stevens, CMT	33
Victoria hospital, Kirkcaldy	David Griffith, Consultant	32
Victoria hospital, Kirkcaldy	Naomi Bulteel, SpR	32
Northumbria Healthcare NHS Foundatio Trust	Charlotte Milne, SpR	30
Northumbria Healthcare NHS Foundatio Trust	Jayanta Sarma, Consultant	30
Ninewells hospital, Dundee	Aline Wilson, SpR	29
Ninewells hospital, Dundee	John Shone, Consultant	29
Ninewells hospital, Dundee	Lynn Urquhart, Consultant	29
Ninewells hospital, Dundee	Sahar Eldirdiri, SpR	29
Royal Preston Hospital, Lancashire Teaching Hospitals NHS Foundation Trust	Alison Muir, Consultant	28
Royal Preston Hospital, Lancashire Teaching Hospitals NHS Foundation Trust	Leila White, Clinical Scientist	28
Sheffield teaching Hospitals	Jody Aberdein, Consultant	28
Sheffield teaching Hospitals	Phillip Simpson, SpR	28
Shrewbury and Telford Hospital NHS Trust	Hnin Hay Mar	26
Shrewbury and Telford Hospital NHS Trust	John Bowen	26
Shrewbury and Telford Hospital NHS Trust	Keying Tan	26
Shrewbury and Telford Hospital NHS Trust	Eint Shwe Zin thein	26
Shrewbury and Telford Hospital NHS Trust	Mahmoud Aziz	26
University Hospital North Midlands	Anthony Cadwgan, Consultant	25
University Hospital North Midlands	Brendan Davies, Consultant	25
University Hospital North Midlands	Daniel White, SpR	25
University Hospital North Midlands	Natasha Weston, SpR	25
University Hospital North Midlands	Salman Zeb, CMT	25
St George's Hospital, London	Angela Houston, Consultant	24
St George's Hospital, London	Imogen Fordham, clinical fellow	24
St George's Hospital, London	Terry John Evans, SpR	24
St George's Hospital, London	Louise Wootton, Physician's associate	24
Nottingham University Hospitals NHS Trust	David Turner, Consultant	24
Nottingham University Hospitals NHS Trust	Iona Willingham, SpR	24
Birmingham Queen Elizabeth Hospital	Aimee Johnson, SpR	23
Birmingham Queen Elizabeth Hospital	Nimal Wickramasinghe, Consultant	23
Salford Royal Infirmary, Salford	Ashley Horsley, SpR	23
Salford Royal Infirmary, Salford	Eamonn Trainor, Consultant	23
Salford Royal Infirmary, Salford	Olivier Gaillemin, Consultant	23
University Hospital Southampton NHS Foundation Trust	Andrew Rosser, Consultant	23
University Hospital Southampton NHS Foundation Trust	Nicholas J Norton, SpR	23
Royal Blackburn Hospital, East Lancashire Hospitals NHS Trust	Iain Crossingham, Consultant	22
Royal Blackburn Hospital, East Lancashire Hospitals NHS Trust	Katie Cheung, Medical Student	22
Royal Blackburn Hospital, East Lancashire Hospitals NHS Trust	Megan Duxbury, CMT	22
Queen Elizabeth University Hospital, NHS Greater Glasgow and Clyde	Ashutosh Deshpande, Consultant	22
Queen Elizabeth University Hospital, NHS Greater Glasgow and Clyde	Emilie Bellhouse, FY2	22
Queen Elizabeth University Hospital, NHS Greater Glasgow and Clyde	Kamaljit Khalsa, SpR	22

For peer review only

Imperial College School of Medicine	Helena Brezovjakova, Medical Student	22
Imperial College School of Medicine	Emma McLean, medical student	22
Imperial college healthcare NHS trust	Tanmay, Kanitkar, CMT	22
Imperial college healthcare NHS trust	Nicholas Davies, Consultant	22
Imperial College School of Medicine	Alexsander Dawidziuk, Medical Student	22
St James University hospital, Leeds	Eloisa Mclaughlin, Medical student	22
St James University hospital, Leeds	Joanna Allen, Consultant	22
St James University hospital, Leeds	Razan Saman, SpR	22
St James University hospital, Leeds	Sarah Kelly, SpR	22
Royal Liverpool University Hospital, Liverpool	Hugh Adler, SpR	22
Royal Liverpool University Hospital, Liverpool	Sylviane Defres, Consultant	22
Arrowe Park Hospital, Wirral	David Harvey, Consultant	21
Arrowe Park Hospital, Wirral	Elshadai Ejere, FY2	21
Queen's hospital, Romford	Aarti Shah, Consultant	21
Queen's hospital, Romford	Yiwen Soo, FY1	21
Raigmore Hospital, Inverness	Wendy Beadles, Consultant	21
Raigmore Hospital, Inverness	Heather Sturgeon, Medical student	21
Raigmore Hospital, Inverness	Brodie Cameron, Medical Student	21
James Cook University Hospital, Middlesbrough	Ben Tomlinson, SpR	20
James Cook University Hospital, Middlesbrough	David Chadwick, Consultant	20
University Hospital Monklands	Claire McGoldrick, Consultant	20
University Hospital Monklands	Katie McDowell, FY2	20
Cumberland infirmary, Carlisle	Alastair Miller, Consultant	19
Cumberland infirmary, Carlisle	Clive Graham, Consultant	19
Cumberland infirmary, Carlisle	Mpho Molosiwa, FY2	19
Newcastle Upon Tyne NHS Foundation Trust	Ewan Hunter, Consultant	19
Newcastle Upon Tyne NHS Foundation Trust	Ruth Owen, Medical Student	19
Newcastle Upon Tyne NHS Foundation Trust	Katherine Flack	19
Airedale hospital, Airedale	Adrian Kennedy, Consultant	18
Bradford Royal Infirmary, Bradford	Amy Robinson, Consultant	16
Bradford Royal Infirmary	Phoebe Cross, SpR	16
Bradford Royal Infirmary	Fay Perry	16
University Hospital Wales	Vithusha Inpadhas	16
Aberdeen Royal Infirmary	Ali Khan, SpR	15
Aberdeen Royal Infirmary	Sarathy Selvam, FY2	15
Aberdeen Royal Infirmary	Vhairi Bateman, Consultant	15
Aberdeen Royal Infirmary	Jeremy Wong, Medica Student	15
Lancaster Royal Infirmary	Henry Wu, FY2	15
Lancaster Royal Infirmary	Monika Pasztor, Consultant	15
Whittington Hospital, London	Trupti Patel, Consultant	14
Whittington Hospital, London	Ajanthiha Karunakaran, Medical Student	14
Russells Hall Hospital, Dudley	Basma Soliman, CT1	13
Russells Hall Hospital, Dudley	Hassan Paraiso, Consultant	13
Glasgow Royal Infirmary	Mairi McLeod, Consultant	13
Glasgow Royal Infirmary	Su su Htwe, SpR	13
Glasgow Royal Infirmary	Anna Smith	13

For peer review only

James Paget University Hospitals NHS Foundation Trust	Andrew Blanshard, CMT	12
James Paget University Hospitals NHS Foundation Trust	Harish Reddy, Consultant	12
Portsmouth Hospitals University NHS Trust	Avneet Shahi, SpR	12
Portsmouth Hospitals University NHS Trust	Helen Chesterfield, Consultant	12
Portsmouth Hospitals University NHS Trust	Oliver Bannister, CMT	12
Withybush hospital, Haverford West	Ben Schroeder, Medical Student	12
Withybush hospital, Haverford West	Ken Woodhouse, Consultant	12
Ashford and St Peter's NHS Foundation Trust	Jan Coebergh, Consultant	11
Ashford and St Peter's NHS Foundation Trust	Viva Levee, FY2	11
Mater Misericordiae University Hospital, Dublin	Eavan Muldoon, Consultant	11
Mater Misericordiae University Hospital, Dublin	Rhea O'regan, SPR	11
Mater Misericordiae University Hospital, Dublin	Tee Keat Teoh, SpR	11
Newham Hospital, Barts Health NHS Trust	Sathyavani Subbarao, SpR	11
Newham Hospital, Barts Health NHS Trust	Simon Tiberi, Consultant	11
Newham Hospital, Barts Health NHS Trust	Caryn Rosmarin	11
London UCL and Hospital for Tropical diseases at University College London Hospitals NHS Foundation Trust.	Jayne Ellis, SpR	10
London UCL and Hospital for Tropical diseases at University College London Hospitals NHS Foundation Trust.	Lucy Bell, CMT	10
London UCL and Hospital for Tropical diseases at University College London Hospitals NHS Foundation Trust.	Robert Heyderman, Consutant	10
Barts Health NHS Trust	Jonathan Lambourne, Consultant	10
Barts Health NHS Trust	Emma McGuire, SpR	10
Barts Health NHS Trust	Robert Serafino, Consultant	10
Guy's and St Thomas' NHS Foundation Trust	Anna Goodman, Consultant	9
Guy's and St Thomas' NHS Foundation Trust	Ishaan Bhide, FY1	
Guy's and St Thomas' NHS Foundation Trust	Karanjeet Sagoo, Medical Student	
Whipps Cross, Barte Health NHS Trust	Mark Melzer, Consultant	8
Whipps Cross, Barte Health NHS Trust	Maria Krutikov, SpR	8
The Royal Free Hospital, London	Indran Balakrishnan, Consultant	6
The Royal Free Hospital, London	Susan Hopkins, Consultant	6
The Royal Free Hospital, London	Tim Jones, SpR	6
Trafford General Hospital, Manchester University NHS Foundation Trust	Kajal Patel, Medical Student	4
Trafford General Hospital, Manchester University NHS Foundation Trust	Barzo Faris, Consultant	
William Harvey Hospital, East Kent	Graeme Calv er, Consultant	3
William Harvey Hospital, East Kent	Ricky Singh, Medical Student	3
William Harvey Hospital, East Kent	Hazel Sanghvi, Medical Student	3
Tameside General Hospital	Mohamed Eltayeb, Clinical Fellow	2
Tameside General Hospital	Rathur Haris, Consultant	2

For peer review only

References

1. van de Beek D, de Gans J, Spanjaard L, Weisfelt M, Reitsma JB, Vermeulen M. Clinical Features and Prognostic Factors in Adults with Bacterial Meningitis. N Engl J Med. 2004;351:1849-59.
2. Okike IO, Ribeiro S, Ramsay M, Heath PT, Sharland M, Ladhani SN. Trends in bacterial, mycobacterial and fungal meningitis in England and Wales 2004-11: an observational study. Lancet Infect Dis. 2014;14:301-7.
3. McGill F, Griffiths MJ, Bonnett LJ, Geretti AM, Michael BD, Beeching NJ, et al. Incidence, aetiology, and sequelae of viral meningitis in UK adults: a multicentre prospective observational cohort study. Lancet Infect Dis. 2018;18(9):992-1003.
4. Proulx N, Frechette D, Toye B, Chan J, Kravcik S. Delays in the administration of antibiotics are associated with mortality from acute bacterial meningitis. QJM. 2005;98:291-8.
5. Bodilsen J, Dalager-Pedersen M, Schonheyder HC, Nielsen H. Time to antibiotic therapy and outcome in bacterial meningitis: a Danish population-based cohort study. BMC Infect Dis. 2016;16(392):1-7.
6. McGill F, Heyderman RS, Michael BD, Defres S, Beeching NJ, Borrow R, et al. The UK joint specialist societies guideline on the diagnosis and management of acute meningitis and meningococcal sepsis in immunocompetent adults. J Infect. 2016;72(4):405-38.
7. Stockdale AJ, Weekes MP, Aliyu SH. An audit of acute bacterial meningitis in a large teaching hospital 2005-10. QJM. 2011;104(12):1055-63.
8. Cullen MM. An audit of the investigation and initial management of adults presenting with possible bacterial meningitis. J Infect. 2005;50:120-4.
9. Brouwer M, van de Beek D. Viral meningitis in the UK: time to speed up. Lancet Infectious Diseases. 2018;18(9):930-1.
10. Kadambari S, Okike I, Ribeiro S, Ramsay ME, Heath PT, Sharland M, et al. Seven-fold increase in viral meningo-encephalitis reports in England and Wales during 2004-2013. J Infect. 2014;69(4):326-32.
11. Glimåker M, Johansson B, Grindborg Ö, Bottai M, Lindquist L, Sjölin J. Adult Bacterial Meningitis: Earlier Treatment and Improved Outcome Following Guideline Revision Promoting Prompt Lumbar Puncture. Clin Infect Dis. 2015;60(8):1162-9.
12. Public Health England. UK Standards for Microbiology Investigations. Investigation of Cerebrospinal fluid. 2017.
13. The Kings Fund. NHS hospital bed numbers: past, present, future 2021 [Available from: https://www.kingsfund.org.uk/publications/nhs-hospital-bed-numbers.
14. Auburtin M, Wolff M, Charpentier J, Varon E, Tulzo YL, Girault C, et al. Detrimental role of delayed antibiotic administration and penicillin-nonsusceptible strains in adult intensive care unit patients with pneumococcal meningitis: The PNEUMOREA prospective multicenter study. Crit Care Medicine. 2006;34:2758-65.
15. Michael B, Menezes BF, Cunniffe J, Miller A, Kneen R, Francis G, et al. Effect of delayed lumbar punctures on the diagnosis of acute bacterial meningitis in adults. Emerg Med J. 2010;27(6):433-8.
16. Costerus JM, Brouwer MC, Sprengers MES, Roosendaal SD, van der Ende A, van de Beek D. Cranial Computed Tomography, Lumbar Puncture, and Clinical Deterioration in Bacterial Meningitis: A Nationwide Cohort Study. . Clin Infect Dis. 2018;67(6):920-6.
17. Costerus JM, Brouwer MC, van de Beek D. Technological advances and changing indications for lumbar puncture in neurological disorders. Lancet Neurol 2018;17(3):268-78.
18. Glimaker M, Johansson B, Bell M, Ericsson M, Blackberg J, Brink M, et al. Early Lumbar Puncture in adult bacterial meningitis - rationale for revised guidelines. Scand J Infect Dis. 2013;45:657-63.
19. Defres S, Mayer J, Backman R, kneen R. Performing lumbar punctures for suspected CNS infections: experience and practice of trainee doctors. Br J Hosp Med. 2015;76(11):658-62.

For peer review only - http://bmjopen.bmj.com/site/about/guidelines.xhtml

20. Heinsbroek E, Ladhani SN, Gray S, Guiver M, Kaczmarski E, Borrow R, et al. Added value of PCR testing for confirmation of invasive meningococcal disease in England. J Infect. 2013;67:385-90.
21. Bouzid D, Zanella MC, Kerneis S, Visseaux B, May L, Schrenzel J, et al. Rapid diagnostic tests for infectious diseases in the emergency department. Clinical microbiology and infection : the official publication of the European Society of Clinical Microbiology and Infectious Diseases. 2020.
22. Giuleri SG, Chapuis-Taillard C, Manuel O, Hugli O, Pinget C, Wasserfallen JB, et al. Rapid detection of enterovirus in cerebrospinal fluid by a fully automated PCR assay is associated with improved management of aseptic meningitis in adult patients. J Clin Virol. 2015;62:58-62.
23. de Gans J, van de Beek D, European Dexamethasone in Adulthood Bacterial Meningitis Study Investigators. Dexamethasone in Adults with Bacterial Meningitis. N Engl J Med. 2002;347(20):1549-56.

For peer review only

447

For peer review only

Supplementary table.

Supplementary table . Risk factors for Listeria stratified by aetiology.

	Total cohort N (%)	*Bacterial meningitis N (%)*	*Viral meningitis N (%)*	*Other meningitis† N (%)*	*P value*
**N**	1,471 (100)	302 (21)	615 (42)	553 (38)	-
Age >60 years	235 (16)	126 (42)	27 (4)	79 (14)	<0.001
*Number immunocompromised by disease/medication**	60 (4)	14 (5)	18 (3)	28 (5)	0.23
Number with Diabetes mellitus	64 (4)	30 (10)	11 (2)	22 (4)	<0.001
Number with a history of alcohol excess	36 (2)	21 (7)	3 (0.5)	12 (2)	<0.001

†= other meningitis category included all patients without a confirmed bacterial or viral pathogen

*=Conditions listed as immunocompromising conditions included haematological malignancy (n=8), Other malignancy (n=8), solid organ transplant (n=6), liver cirrhosis (n=1), HIV (n=9), Pregnancy (n=2). Medicaion listed included Steroids (n=7), tocilizumab, ecolizumab and infliximab (n=6), Methotrexate (n=8), Mycophenolate (n=2), Azathioprine (n=3), 'chemotherapy' (n=4). (some patients had more than one immunocompromising condition/medication).

For peer review only

STROBE Statement—Checklist of items that should be included in reports of ***cohort studies***

	Item No	Recommendation	Location in paper
**Title and abstract**	1	(*a*) Indicate the study's design with a commonly used term in the title or the abstract	Title, page 1, line 1
		(*b*) Provide in the abstract an informative and balanced summary of what was done and what was found	Abstract, page 2, line 40 onwards
**Introduction**			
Background/rationale	2	Explain the scientific background and rationale for the investigation being reported	Introduction, page 4 line 80 onwards
Objectives	3	State specific objectives, including any prespecified hypotheses	Introduction, page 5, line 104
**Methods**			
Study design	4	Present key elements of study design early in the paper	Methods, page 5, line 108 onwards
Setting	5	Describe the setting, locations, and relevant dates, including periods of recruitment, exposure, follow-up, and data collection	Methods, page 5, line 108 onwards
Participants	6	(*a*) Give the eligibility criteria, and the sources and methods of selection of participants. Describe methods of follow-up	Methods, page 6, box 1
		(*b*) For matched studies, give matching criteria and number of exposed and unexposed	NA
Variables	7	Clearly define all outcomes, exposures, predictors, potential confounders, and effect modifiers. Give diagnostic criteria, if applicable	Methods, page 6, box 1 and line 125 onwards
Data sources/ measurement	8*	For each variable of interest, give sources of data and details of methods of assessment (measurement). Describe comparability of assessment methods if there is more than one group	Methods, page 5, line 110 onwards
Bias	9	Describe any efforts to address potential sources of bias	Methods, page 5, line 117 onwards Discussion, page 17, line 294 onwards
Study size	10	Explain how the study size was arrived at	Methods, page 5, line 110 onwards
Quantitative variables	11	Explain how quantitative variables were handled in the analyses. If applicable, describe which groupings were chosen and why	Methods, page 7, line 141 onwards
Statistical methods	12	(*a*) Describe all statistical methods, including those used to control for confounding	Methods, page 7, line 141 onwards
		(*b*) Describe any methods used to examine subgroups and interactions	NA
		(*c*) Explain how missing data were addressed	Methods, page 6, line 130
		(*d*) If applicable, explain how loss to follow-up was addressed	NA
		(*e*) Describe any sensitivity analyses	NA

Results

For peer review only - http://bmjopen.bmj.com/site/about/guidelines.xhtml

Participants	13*	(a) Report numbers of individuals at each stage of study—eg numbers potentially eligible, examined for eligibility, confirmed eligible, included in the study, completing follow-up, and analysed	Results, page 7, line 156
		(b) Give reasons for non-participation at each stage	NA
		(c) Consider use of a flow diagram	NA
Descriptive data	14*	(a) Give characteristics of study participants (eg demographic, clinical, social) and information on exposures and potential confounders	Page 9, Table 1
		(b) Indicate number of participants with missing data for each variable of interest	Page 10, Table 2
		(c) Summarise follow-up time (eg, average and total amount)	NA
Outcome data	15*	Report numbers of outcome events or summary measures over time	Page 9 Table 1, page 10, table 2 and page 15, table 3
Main results	16	(*a*) Give unadjusted estimates and, if applicable, confounder-adjusted estimates and their precision (eg, 95% confidence interval). Make clear which confounders were adjusted for and why they were included	Results, page 14, line 230 onwards
		(*b*) Report category boundaries when continuous variables were categorized	NA
		(*c*) If relevant, consider translating estimates of relative risk into absolute risk for a meaningful time period	NA
Other analyses	17	Report other analyses done—eg analyses of subgroups and interactions, and sensitivity analyses	NA
**Discussion**			
Key results	18	Summarise key results with reference to study objectives	Discussion, page 16 line 249 onwards
Limitations	19	Discuss limitations of the study, taking into account sources of potential bias or imprecision. Discuss both direction and magnitude of any potential bias	Discussion, page 17, line 294 onwards
Interpretation	20	Give a cautious overall interpretation of results considering objectives, limitations, multiplicity of analyses, results from similar studies, and other relevant evidence	Conclusion, page 18, line 313 onwards
Generalisability	21	Discuss the generalisability (external validity) of the study results	Discussion, page 17 line 296-8
**Other information**			
Funding	22	Give the source of funding and the role of the funders for the present study and, if applicable, for the original study on which the present article is based	Page 22, Line 439

*Give information separately for exposed and unexposed groups.

Note: An Explanation and Elaboration article discusses each checklist item and gives methodological background and published examples of transparent reporting. The STROBE checklist is best used in conjunction with this article (freely available on the Web sites of PLoS Medicine at http://www.plosmedicine.org/, Annals of Internal Medicine at http://www.annals.org/, and Epidemiology at http://www.epidem.com/). Information on the STROBE Initiative is available at http://www.strobe-statement.org.

For peer review only - http://bmjopen.bmj.com/site/about/guidelines.xhtml
For peer review only

For peer review only

For peer review only - http://bmjopen.bmj.com/site/about/guidelines.xhtml

BMJ Open

Clinical management of community-acquired meningitis in adults in the United Kingdom and Ireland in 2017: a retrospective cohort study on behalf of the National Infection Trainees Collaborative for Audit and Research (NITCAR)

Journal:	*BMJ Open*
Manuscript ID	bmjopen-2022-062698.R1
Article Type:	Original research
Date Submitted by the Author:	31-May-2022
Complete List of Authors:	Ellis, Jayne; UCL,<br>Harvey, David; Wirral University Teaching Hospital NHS Foundation Trust<br>Defres, Sylviane; University of Liverpool; Royal Liverpool and Broadgreen Hospitals NHS Trust<br>Chandna, Arjun ; Cambodia Oxford Medical Research Unit, Angkor Hospital for Children, ; University of Oxford Centre for Tropical Medicine and Global Health,<br>MacLachlan, Eloisa; University of Leeds; National Student Association of Medical Research<br>Solomon, Tom; University of Liverpool, Neurological Science, Medical Microbiology<br>Heyderman, Robert; University College London, Division of Infection and Immunity; University of Malawi College of Medicine, Malawi Liverpool Wellcome Trust Clinical Research Programme<br>McGill, Fiona; University of Liverpool, Institute of Infection and Global Health; Leeds Teaching Hospitals NHS Trust, Infectious Diseases<br>study group, NAMM; British Infection Association, Collaborative authorship group
**Primary Subject Heading**:	Infectious diseases
Secondary Subject Heading:	Emergency medicine, Neurology
Keywords:	Diagnostic microbiology < INFECTIOUS DISEASES, Molecular diagnostics < INFECTIOUS DISEASES, Infectious disease/HIV < NEUROLOGY, INTERNAL MEDICINE, Protocols & guidelines < HEALTH SERVICES ADMINISTRATION & MANAGEMENT, BACTERIOLOGY

SCHOLARONE™
Manuscripts

I, the Submitting Author has the right to grant and does grant on behalf of all authors of the Work (as defined in the below author licence), an exclusive licence and/or a non-exclusive licence for contributions from authors who are: i) UK Crown employees; ii) where BMJ has agreed a CC-BY licence shall apply, and/or iii) in accordance with the terms applicable for US Federal Government officers or employees acting as part of their official duties; on a worldwide, perpetual, irrevocable, royalty-free basis to BMJ Publishing Group Ltd ("BMJ") its licensees and where the relevant Journal is co-owned by BMJ to the co-owners of the Journal, to publish the Work in this journal and any other BMJ products and to exploit all rights, as set out in our licence.

The Submitting Author accepts and understands that any supply made under these terms is made by BMJ to the Submitting Author unless you are acting as an employee on behalf of your employer or a postgraduate student of an affiliated institution which is paying any applicable article publishing charge ("APC") for Open Access articles. Where the Submitting Author wishes to make the Work available on an Open Access basis (and intends to pay the relevant APC), the terms of reuse of such Open Access shall be governed by a Creative Commons licence – details of these licences and which Creative Commons licence will apply to this Work are set out in our licence referred to above.

Other than as permitted in any relevant BMJ Author's Self Archiving Policies, I confirm this Work has not been accepted for publication elsewhere, is not being considered for publication elsewhere and does not duplicate material already published. I confirm all authors consent to publication of this Work and authorise the granting of this licence.

For peer review only

Clinical management of community-acquired meningitis in adults in the United Kingdom and Ireland in 2017: a retrospective cohort study on behalf of the National Infection Trainees Collaborative for Audit and Research (NITCAR)

Jayne Ellis, Specialty trainee[1], David Harvey, Consultant[2], Sylviane Defres, Consultant[3,4,10], Arjun Chandna, Specialty trainee[5,6], Eloisa Maclachlan, Medical Student[7,8], Tom Solomon, Professor[4,9,10], Robert S Heyderman (0000-0003-4573-449X), Professor[1,11], Fiona McGill (0000-0002-0903-9046), Consultant[4,10,12] on behalf of the National Audit of Meningitis Management (NAMM) group

1. Hospital for Tropical Diseases, University College London Hospitals NHS Foundation Trust, London, UK.
2. Wirral University Teaching Hospital NHS Foundation Trust, UK.
3. Liverpool University Hospitals NHS Foundation Trust, Liverpool, UK.
4. Institute of Infection, Veterinary and Ecological Sciences, University of Liverpool, Liverpool, UK.

Nuffield department of Medicine, University of Oxford, Oxford, UK.

5. Department of Clinical Research, London School of Hygiene and Tropical Medicine, London, UK.
6. Mahidol-Oxford Tropical Medicine Research Unit, Mahidol University, Bangkok, Thailand.
7. University of Leeds, Leeds, UK.
8. National Student Association for Medical Research.
9. The Walton Centre NHS Foundation Trust, Liverpool, UK
10. NIHR Health Protection Research Unit for Emerging and Zoonotic Infections, University of Liverpool, Liverpool, UK.
11. Research Department of Infection, Division of Infection and Immunity, University College London, London, UK.
12. Department of Infection and Travel Medicine and Department of Microbiology, Leeds Teaching Hospitals NHS Trust, Leeds, UK.

Correspondence to: Dr Fiona McGill, Leeds Teaching Hospitals NHS Trust, Beckett Street, Leeds, LS9 7TF

f.mcgill@nhs.net

Word count: 2901

For peer review only

For peer review only - http://bmjopen.bmj.com/site/about/guidelines.xhtml

Abstract

Objectives: To assess practice in the care of adults with suspected community acquired bacterial meningitis in the UK and Ireland.

Design: Retrospective cohort study.

Setting: 64 UK and Irish hospitals.

Participants: 1,471 adults with community acquired meningitis of any aetiology in 2017.

Results: None of the audit standards, from the 2016 UK Joint Specialists Societies guideline on diagnosis and management of meningitis, were met in all cases. With respect to 20 of 30 assessed standards, clinical management provided for patients was in line with recommendations in less than 50% of cases. 45% of patients had blood cultures taken within an hour of admission, 0.5% had a lumbar puncture within one hour, 26% within 8 hours. 28% had bacterial molecular diagnostic tests on CSF. Median time to first dose of antibiotics was 3.2 hours (IQR 1.3-9.2). 82% received empirical parenteral cephalosporins. 55% ≥ 60 years and 31% of immunocompromised patients received anti-*Listeria* antibiotics. 21% received steroids. Of the 1,471 patients, 21% had confirmed bacterial meningitis. Amongst those with bacterial meningitis, pneumococcal aetiology, admission to intensive care and initial Glasgow Coma Scale score less than 14 were associated with in-hospital mortality (adjusted odds ratio [aOR] 2.08, 95% CI 0.96 – 4.48; aOR 4.28, 95% CI 1.81 – 10.1; aOR 2.90, 95% CI 1.26 – 6.71 respectively). Dexamethasone therapy was weakly associated with a reduction in mortality in both those with proven bacterial meningitis (aOR 0.57, 95% CI 0.28 – 1.17) and with pneumococcal meningitis (aOR 0.47, 95% CI 0.20 – 1.10).

Conclusion: This study demonstrates that clinical care for patients with meningitis in the UK is not in line with current evidence-based national guidelines. Diagnostics and therapeutics should be targeted for quality improvement strategies. Work should be done to improve the impact of guidelines, understand why they are not followed and, once published, ensure they translate into changed practice.

For peer review only

For peer review only - http://bmjopen.bmj.com/site/about/guidelines.xhtml

Keywords: meningitis, adults, bacteria, antibiotics, management, guidelines

For peer review only

Strengths and limitations of this study

- To our knowledge, this is the largest national study of the management of meningitis in the UK published to date.
- The study includes all suspected community acquired bacterial meningitis, allowing assessment of early clinical care prior to an aetiological diagnosis being made.
- The study is widely translatable and representative of practice within the UK and Ireland.
- The study is limited by its retrospective design which brings associated recall bias and some missing data.
- The study may also be limited by the self-selection of the sites included.

Introduction

Acute bacterial meningitis is a medical emergency associated with considerable death and disability in the UK(1). Successful immunisation programmes targeting *Haemophilus influenzae* type b, *Streptococcus pneumoniae* and *Neisseria meningitidis* means that community acquired bacterial meningitis, particularly in children and adolescents, is now relatively rare (2). The incidence of bacterial meningitis in adults in England is estimated to be approximately 1-1.25 per 100,000 population overall, exceeding 9 per 100,000 in people over 70 years (2, 3).

Early recognition of meningitis, appropriate investigation, and treatment, saves lives (4, 5). It is essential that front-line clinicians, who may not encounter meningitis very often, are vigilant and have a high index of suspicion to minimise poor outcomes. To help staff who are seeing patients with suspected meningitis, the UK guidelines on the diagnosis and management of acute meningitis and meningococcal sepsis in immunocompetent adults were published in 2016(6). The guidelines provide readily accessible, comprehensive, evidenced-based recommendations. Previous studies show that clinical care delivered in the UK is frequently non-adherent to guidelines(7, 8). A more recent UK study highlighted a large amount of inappropriate brain imaging prior to lumbar punctures (LPs) and long delays in performing LPs (3, 9). Inadequate use of molecular diagnostics and HIV-testing have also been

For peer review only

highlighted as areas for improvement(3). The increasing risk of multi-drug resistant bacteria, an ageing population susceptible to a wider variety of bacteria (e.g. *Listeria monocytogenes, Escherichia coli* and *Klebsiella pneumoniae*)(2) and a greater appreciation that viruses are common causes of meningitis(10, 11), make diagnostics essential. Reports from outside the UK have shown improvements in outcomes following guideline publication and implementation (12). We carried out a retrospective observational study with the dual aims of i) assessing current clinical practice regarding diagnosis and management of adult patients with suspected community acquired bacterial meningitis, and ii) to identify areas for improvement.

Methods

Hospitals in the UK were invited to take part in this study via the National Infection Trainees Collaborative for Audit and Research (NITCAR) network, the UK Meningitis study network, the British Infection Association (BIA) and through personal contacts. Eligible patients were identified via hospital coding data, laboratory data, or a combination of both. Data from patients aged 16 or over who presented with suspected acute community acquired bacterial meningitis during 2017 were eligible for screening. Patients who met our case definition for confirmed acute meningitis, regardless of aetiology, were eligible for inclusion (box 1). Definitions are as previously published(3). Many interventions are performed prior to knowing the diagnosis, therefore, we included all meningitis in the analysis, including viral and those in whom no pathogen was identified. This allowed us to assess the entire clinical pathway of patients presenting with possible bacterial meningitis, although some would be ultimately diagnosed with a different aetiology.

For peer review only

Box 1: Inclusion and exclusion criteria for cases of meningitis

A meningitis case was defined as:

(1) Patients with a cerebrospinal fluid (CSF) WCC >4 x10^6 cells/L (regardless of whether a pathogen was identified or not) and a clinical suspicion of meningitis at the time OR

(2) in the case of bacterial meningitis, symptoms and signs of meningitis with a significant pathogen in the CSF (culture or PCR) or blood regardless of CSF leukocyte count.

Patients with the following diagnoses were excluded:

(1) Cryptococcal meningitis;

(2) Tuberculous meningitis;

(3) Nosocomial meningitis (defined as meningitis that occurs during a hospital admission or within 30 days of discharge or meningitis associated with indwelling devices in the central nervous system)

(4) Encephalitis (defined as altered consciousness for >24 with no other cause found and two or more of the following signs: fever or history of fever (≥38°C) during the current illness; seizures or focal neurological signs (with evidence of brain parenchyma involvement); CSF pleocytosis (>4 × 10^6 cells per L); EEG suggesting encephalitis; and neuroimaging suggestive of encephalitis).

Standards indicative of good practice were taken from the 2016 UK Joint Specialists Societies guideline on the diagnosis and management of meningitis and meningococcal sepsis in immunocompetent adults, and the Standards in Microbiological Investigations on the processing of cerebrospinal fluid (B27) (6, 13). For each standard, the number of patients as a proportion of the total cohort who received clinical care in line with the standard is reported. A second adjusted analysis taking account of missing data is also reported, whereby the number of patients as a proportion of the cohort with available data who received clinical care in line with the standard was reported.

For peer review only - http://bmjopen.bmj.com/site/about/guidelines.xhtml

Data were collected using electronic case report forms on REDcap™, a password protected central web-based database system. All microbiological diagnostic procedures were performed at the local hospital laboratory for each participating site using locally approved procedures. All data were anonymised and recorded under a unique participant identification number.

Ethics approval

As all data were anonymised individual patient consent and ethical approval was not required. The study was registered with each site's clinical governance department in line with local procedure.

Statistical analyses

Descriptive statistics were used to summarize data. Categorical data were summarized using counts and percentages. Denominators presented are based on available data, where incomplete case records were submitted by contributing sites. For continuous variables, means and ranges or medians and inter-quartile ranges (IQRs) are presented depending on the distribution of the data. Categorical data were analysed using Chi squared or Fisher's exact test. Continuous data were analysed using t-tests, Mann Whitney U or Kruskall Wallis depending on the distribution of the data. Regression analysis was used to identify potential risk factors associated with poor outcomes.

Patient and public involvement

Although there was no direct involvement of patients and public in this study the Meningitis Research Foundation, a key advocacy group for patients are represented in the authorship of the original guidelines and will be key in the dissemination of the results and the subsequent call to improve practice. Preliminary results have been shared with the Meningitis Research Foundation and some of their members.

Results

For peer review only

1,471 patients from 64 hospitals throughout the UK and Ireland took part (see appendix). The hospitals ranged in size from small district generals to larger teaching hospitals. The mean number of beds was 846 (range 230-2000). The hospitals who took part in England comprised 45% of the total acute bed base in England, (42,612/94,827(14)). Females accounted for 57% (n=838) and the median age was 34 years (IQR 26,49). Confirmed viral meningitis occurred in 615 (42%) and 303 had confirmed bacterial meningitis (21%). More than one third of patients (n=553) fulfilled the case definition (box 1) but had no confirmed microbiological diagnosis and were therefore categorised as meningitis of unknown aetiology. Using the criteria proposed by Spanos et al(15) 56 of those without a confirmed aetiology could be assumed to have bacterial meningitis. *Streptococcus pneumoniae* and *Neisseria meningitidis,* were the most common bacterial pathogens, where a cause was found, accounting for 172 (57%) and 76 (25%) of cases respectively. *Haemophilus influenzae* (serotypes unknown) was found in 14 cases. *Enteroviruses* were the most common viral pathogens occurring in 429 (69%) of all confirmed viral meningitis. *Herpes simplex virus-2* was the second most common viral pathogen detected in 97 (16%) of viral cases. Baseline demographics and clinical characteristics are shown in table 1.

For peer review only

Table 1: Baseline demographics, timing of key investigations and clinical outcomes of 1,471 adults presenting with suspected meningitis

	Total cohort N (%)	*Bacterial meningitis N (%)*	*Viral meningitis N (%)*	*Other† N (%)*	*P value[1]*
**N**	1,471 (100)	303 (21)	615 (42)	553 (38)	-
**Median age (IQR)**	34 (26 – 49)	54 (36 – 65)	31 (25 – 37)	34 (26 – 48)	< 0.001
**Male**	625 (43)	173 (57)	214 (35)	238 (43)	< 0.001
**In patient mortality**	48 (3)	38 (13)	2 (0.3)	8 (1.4)	< 0.001
**ITU admission**	192 (13)	157 (53)	4 (0.7)	31 (6)	< 0.001
Median Admission GCS (IQR)	15 (14-15)	13 (9 – 15)	15 (15-15)	15 (15-15)	< 0.001
**Median time (hours) from admission to first antibiotics (IQR)**	2.7 (0.9 – 8.3)	1.5 (0.4 – 5.3)	3.2 (1.3 – 8.3)	3.3. (1 – 12.5)	< 0.001
**Median time (hours) from admission to blood cultures (IQR)**	1 (0.3 – 4)	0.7 (0.2 – 2.4)	1 (0.3 – 3.7)	1.4 (0.3 – 6.1)	0.003
**CT head prior to LP**	1,094 (94)	207 (93)	459 (94)	428 (95)	0.55
**Median time (hours) from admission to LP (IQR)**	16.4 (7.9 – 26.7)	14.8 (7.7 – 29.8)	14.3 (7.5 – 22.6)	20 (8.8 – 35.8)	< 0.001
**Adjunctive dexamethasone**	300 (21)	150 (50)	69 (11)	81 (15)	< 0.001
Median CSF leucocyte count (IQR)	140 (44-399)	930 (235.5 – 3062.5)	122 (48 – 276)	85 (26.8 – 250.3)	< 0.001
Median CSF protein (IQR)	0.68 (0.46 - 1.21)	3.25 (1.4 – 5.8)	0.63 (0.45-0.9)	0.6 (0.4-1.0)	< 0.001
Median CSF glucose (IQR)	3.2 (2.8-3.7)	2.1 (0.95 – 3.45)	3.2 (2.9 – 3.6)	3.3 (3.0-3.8)	< 0.001

†= other meningitis category included all patients without a confirmed bacterial or viral pathogen
1 = for continuous variables, the Kruskal-Wallis test was used to compare medians across groups, and for categorical variables Chi squared tests were used.

Adherence to specific standards of good practice is shown in table 2. None were adhered to 100% of the time. Two thirds of the standards (n=20) had less than or equal to 50% adherence.

For peer review only

Table 2: Adherence to audit standards^				
**Immediate management**				
	**Number achieved standard/total number of patients analysed**	**% of total**	**Number achieved standard/total number of patients evaluable***	**% of number evaluable**
1. The patient's conscious level should be documented using the Glasgow coma scale.	1,283/1,471	87%	1283/1448	89%
2. Blood cultures should be taken as soon as possible and within 1 h of arrival at hospital	326/1,471[1]	22%	326/767[2]	42%
3. LP should be performed within 1 h of arrival at hospital provided that it is safe to do so	8/1,471[3]	0.5%	8/1379[4]	0.6%
4. Antibiotic treatment should be commenced within the first hour	207/1471[5]	14%	207/1083[6]	19%
5. Patients with meningitis and meningococcal sepsis should be cared for with the input of an infection specialist such as a microbiologist or a physician with training in infectious diseases and/or microbiology	1,148/1,471[7]	78%	1148/1464	78%
**Investigations**				
6. Blood culture should be sent	977/1,471	66%	977/1469	67%
7. Blood Pneumococcal PCR should be sent	211/1,471	14%	211/1460	14%
8. Blood Meningococcal PCR should be sent	232/1,471	16%	232/1461	16%
9. CSF opening pressure should be documented	655/1,428[8]	46%	655/1361[9]	48%
10. CSF glucose with concurrent plasma glucose should be sent	607/1,428[8]	43%	607/1415	43%
11. CSF protein should be sent	1,358/1,428[8]	95%	1358/1420	96%
12. Microscopy of the CSF should take place within 2 hours of the lumbar puncture	596/1428[8]	42%	596/1203[10]	50%
13. CSF for pneumococcal PCR should be sent in all cases of suspected bacterial meningitis	412/1,428[8]	29%	412/1418	29%
14. CSF for Meningococcal PCR should be sent in all cases of suspected bacterial meningitis	434/1,428[8]	30%	434/1418	31%
15. A swab of the posterior nasopharyngeal wall should be obtained as soon as possible, and sent for meningococcal culture, in all cases of suspected meningococcal meningitis/sepsis	54/1,471	4%	54/1463[11]	4%
16. All patients with meningitis should have an HIV test	646/1,471	44%	646/1459[12]	44%
**Treatment**				
17. All patients with suspected meningitis or meningococcal sepsis should be given ceftriaxone or cefotaxime	1039/1471[13]	71%	1039/1423[14]	73%
18. If the patient has, within the last 6 months, been to a country where penicillin resistant pneumococci are prevalent, IV vancomycin 15-20 mg/kg should be added 12-hourly (or 600 mg rifampicin 12-hourly IV or orally) [15]	See footnote			

For peer review only

19. Those aged 60 or over should receive 2 g IV ampicillin/amoxicillin 4-hourly in addition to a cephalosporin [1B].	55/233	24%	55/197[16]	28%
20. Immunocompromised patients (including diabetics and those with a history of alcohol misuse) should receive 2 g IV ampicillin/amoxicillin 4-hourly in addition to a cephalosporin.	26/115[17]	23%	26/99[18]	26%
21. If there is a clear history of anaphylaxis to penicillins or cephalosporins give IV chloramphenicol 25 mg/kg 6- hourly	14/37	38%	14/30[19]	47%
22. If Streptococcus pneumoniae is identified continue with IV benzylpenicillin 2.4 g 4 hourly, 2 g ceftriaxone IV 12 hourly or 2 g cefotaxime IV 6-hourly	114/172	66%	114/145[20]	79%
23. If N. meningitidis is identified 2 g ceftriaxone IV 12 hourly, 2 g cefotaxime IV 6-hourly or 2.4 benzylpenicillin IV 4-hourly may be given as an alternative	52/76	68%	52/68[21]	76%
24. If the patient is not treated with ceftriaxone (in meningococcal disease), a single dose of 500 mg ciprofloxacin orally should also be given	0/2	0%	0/2	0%
25. If *Listeria monocytogenes* is identified Give 2 g ampicillin/amoxicillin IV 4-hourly and continue for at least 21 days. Co-trimoxazole 10-20 mg/kg or chloramphenicol are alternatives in cases of anaphylaxis to beta lactams.	4/7	57%	4/6	67%[22]
26. If *Haemophilus influenzae* is identified continue 2g ceftriaxone IV 12-hourly or 2 g cefotaxime IV 6-hourly for 10 days	9/14	64%	9/13	69%[23]
27. 10 mg dexamethasone IV 6 hourly should be started on admission, either shortly before or simultaneously with antibiotics.	67/1,471	5%	67/1435[24]	5%
28. If pneumococcal meningitis is confirmed dexamethasone should be continued for 4 days.	34/172[25]	20%	34/158[26]	22%
**Critical Care**				
29. The following patients should be transferred to critical care - those with a rapidly evolving rash, those with a GCS of 12 or less and those with uncontrolled seizures	151/203[27]	74%	151/203	74%
**Notification**				
30. All cases of meningitis (regardless of aetiology) should be notified to the relevant public health authority	236/1,471	16%	236/1465	16%

Notes.

^Only those audit standards that could be measured from the data collected. *excludes those where there was missing data and/or where not relevant. 1. Only 977 patients had blood cultures taken. 2. Excluding those who did not have blood cultures taken and where data was missing. 3. 1428 patients had a LP. 4. Excludes those who did not have an LP and where data was not available. 5. 82 patients had data consistent with having antibiotics prior to admission, this might be due to confusion about whether admission meant admission to the emergency department or admission to a ward, or it may represent data entry error therefore, these figures are not included. 6. 388 patients did not receive any antibiotics at all. 7. 310 (21%) of patients were admitted under an infection specialist, all others received consulting advice only. 8. 43 people did not have an LP. 9. Missing data on 67. 10. 43 had no LP, 97 missing data, 128 time of microscopy was before or at the same time as the LP. 11. Performed in 15/76 (20%) of proven meningococcal cases. 12. 9 known HIV positive and 3 missing data. 13. 285 patients were not given any antibiotics at all. 14. 48 patients who were definitely given antibiotics had missing data on which antibiotics they were given. 15. Using mainland Europe data only and with reference to ECDC data – 101 patients were documented to have travelled to a mainland European country within the previous 6 months. Travel history was not documented at all in 822 cases (56%). Of the 101 patients who had travelled to mainland Europe 54 (54%) had been to a country with a rate of penicillin resistant pneumococci of >5% (2017 data). 5/52 had no antibiotics. 0/47 had antibiotics to cover for penicillin resistant pneumococci. 16. 233 patients were aged over 60 but only 207 received antibiotics. Missing data for 10, 108 received amoxicillin at some point but only 55 received the correct dose. 17. Not including those >=60. 18. 15 did not received any antibiotics and missing data on 1. 19. 7 patients had no antibiotics at all. 20. 27 patients had insufficient antibiotic data. 21. 8 patient had insufficient antibiotic data. 22. 1 patient had insufficient antibiotic data. 23. Insufficient antibiotic data on 1 person. 24. Missing data on 36 – 11 on whether dexamethasone was received or not, 21 on the dose given and 4 on the timing. 25. Only 18 were given the correct dose (10mg). Some received dexamethasone for longer than 4 days. 26. Missing data on 14 individuals. 27. 7/11 patient with progressing rash, 131/176 patients with GCS <13 and 13/16 patients with uncontrolled seizures.

For peer review only

Overall in-hospital mortality was low [48/1471 (3%)]. The mortality was higher in bacterial meningitis (28/302, 13%), and pneumococcal meningitis in particular (28/172, 16%). Mortality in viral meningitis was 0.3% (2/615) and 1.5% (8/548) in those with meningitis of unknown aetiology**.** Just over half (157) of those with confirmed bacterial meningitis required admission to an intensive care unit (ICU).

Use of diagnostics

A few patients, 42, did not have an LP, of whom 26 (62%) had no contraindication (as specified in the 2016 joint specialties guidelines and shown in box 2). Five had meningococcal sepsis without clinical evidence of meningitis. The remaining 37 had clinical symptoms of meningism as well as a positive blood culture (n=35, 83%) and/or a positive blood PCR (n=16, 38%) for either *Streptococcus pneumoniae* (n=23, 55%), *Neisseria meningitidis* (n=18, 43%) or *Listeria monocytogenes* (n=1, 2%).

Box 2: Indications for neuroimaging before lumbar puncture (LP) in suspected meningitis

(1) Focal neurological signs
(2) Presence of papilloedema
(3) Continuous or uncontrolled seizures
(4) GCS ≤ 12

Contra-indications for immediate LP were uncommon and occurred in 299 (20%) patients. Glasgow coma score (GCS) ≤ 12 was the most common contra-indication for immediate LP reported in 143 (10%), followed by focal neurological signs in 38 (3%). A further 70 (7%) had other indications to delay LP. Neuroimaging prior to LP happened in 1094 of 1158 patients (94%), 911 (83%) of whom had no guideline-specified indication. Neuroimaging was performed a median of 11 hours post arrival at hospital (IQR 4-21). Median

For peer review only

time from admission to LP was 16.5 hours (IQR 8 – 27). Only 6 patients had an LP within 1 hour of arrival at hospital and only 326 (26%) within 8 hours.

Median time from LP to CSF microscopy was 2 hours (IQR 1.1-3.2). Time from LP to CSF analysis was significantly quicker when performed at on-site laboratories when compared to centralised laboratory processing (median 1.65 hours (IQR 1.0 - 2.8) compared to 2.95 hours (IQR 2.0 - 3.8) $p < 0.001$).

Fewer than one third of patients had pneumococcal (412, 28%) and meningococcal polymerase chain reaction (PCR) (434, 29.5%) performed on their CSF. Pneumococcal PCR was done on blood in 211 (14%) patients, and meningococcal PCR in 232 (16%). 646 patients (44%) patients had a documented HIV test. Four of these were positive – two of whom had pneumococcal meningitis, one of whom had enteroviral meningitis and one had meningitis of unknown aetiology. Nine patients were previously known to be HIV positive.

Blood cultures were taken from 66% (n=977) of patients with 45% (n=438) having them taken within one hour of arrival at hospital.

Treatment

285 patients (19%) did not receive antibiotics, most of whom had either viral meningitis (163) or lymphocytic meningitis with no aetiology identified (105). The remaining 1,186 patients received at least one dose of antibiotics. The median time from hospital admission to first dose of antibiotics was 3.2 hours (IQR 1.3,9.2). Amongst the patients who received antibiotics the antimicrobials were commenced within an hour of arrival at hospital for approximately one fifth of patients (207/1000). In confirmed bacterial meningitis cases, 92 patients (36%) received antibiotics within an hour of arrival.

Adherence with guideline specified empirical antibiotic regimens was good with 912 (80%) receiving a third-generation cephalosporin. Data is missing on antibiotic type for 47 patients. Of the 197 patients aged 60 years and over who received antibiotics, 108 (55%)

For peer review only

received ampicillin or amoxicillin; only 55 (28%) of those had the correct dose and dosing frequency as recommended for *Listeria monocytogenes* meningitis. Similarly, only 36 (31%) of the immunocompromised patients, who were aged under 60, (n=115) received any ampicillin or amoxicillin for anti-*Listeria* cover. Supplementary table 1 shows details regarding risk factors for Listeria.

Only 300 patients (20%) received adjunctive steroids as recommended. Steroids were given more frequently in patient with confirmed bacterial meningitis in 150 (50%) cases. In patients with pneumococcal meningitis 97 patients (57%) received steroids.

Clinical outcomes

On multivariate analysis, having a confirmed diagnosis of bacterial meningitis was strongly associated with in-hospital mortality. Adjusting for age and sex, confirmed bacterial meningitis was associated with 26 times the odds of in-hospital mortality compared to those with other forms of meningitis (adjusted odds ratio [aOR] 25.9, 95% CI 5.93 – 113.0), including those with no aetiology identified.

In patients with confirmed bacterial meningitis, on univariate analyses, in-hospital mortality was associated with a positive blood culture (crude odds ratio [cOR] 2.21, 95% CI 1.04 – 4.67); GCS $\leq$ 13 (cOR 3.24, 95% CI 1.39 – 7.52), confirmed *Streptococcus pneumoniae* meningitis (cOR 2.37, 95% CI 1.10 – 5.11); and ICU admission (cOR 4.81, 95% CI 1.99 – 11.60). These associations remained despite multivariate adjustment for age and sex (table 3).

The analysis was also conducted using only data from those who had had an LP (supplementary table 2). The association between a positive blood culture and mortality was lost. The association between confirmed pneumococcal aetiology and mortality was approaching statistical significance and the association of ITU admission was maintained.

For peer review only

Table 3: Multivariate analysis of the association between baseline co-variates and in-hospital mortality in 303 patients with confirmed bacterial meningitis using logistic regression modelling

Baseline co-variate	N	In-hospital mortality N (%)[1]	Crude OR for in-hospital mortality (95% CI)	P-value	Adjusted OR for in-hospital mortality (95% CI)*	P-value†
**Sex**						
Male	173	26 (15.1)	1			
Female	130	12 (9.23)	0.57 (0.27-1.18)	0.13		
**Age group**						
≤ 18 years	18	0 (0)				
19 – 59 years	159	18 (11.3)	1			
≥ 60 years	126	20 (16.0)	1.49 (0.75 – 2.96)	0.25		
**Blood culture positive**						
No	137	11 (8.09)	1		1	
Yes	166	27 (16.3)	2.21 (1.04 – 4.67)	0.03	1.87 (0.87 – 4.01)	0.10
**GCS ≤ 13[2]**						
No	124	8 (6.45)	1		1	
Yes	148	27 (18.2)	3.24 (1.39 – 7.52)	0.004	2.90 (1.26 – 6.71)	0.008
**IV dexamethasone given[3]**						
No	149	23 (15.4)	1		1	
Yes	150	14 (9.40)	0.57 (0.27 – 1.16)	0.11	0.57 (0.28 – 1.17)	0.12
**IV dexamethasone given if *Strep.pneumoniae*[4]**						
No	73	16 (21.9)	1		1	
Yes	97	11 (11.5)	0.46 (0.20 – 1.08)	0.07	0.47 (0.20 – 1.10)	0.08
**Final diagnosis *S. pneumoniae***						
No	131	10 (7.63)	1		1	
Yes	172	28 (16.4)	2.37 (1.10 – 5.11)	0.02	2.08 (0.96 – 4.48)	0.05
**ITU admission[5]**						
No	144	7 (4.86)	1		1	
Yes	157	31 (19.7)	4.81 (1.99 – 11.60)	< 0.001	4.28 (1.81 – 10.1)	< 0.001

*adjusted for sex and age group
† P-value from LRT comparing models with and without primary exposure variable
1 = One participant had missing outcome data
2 = 31/303 (10%) participants did not have a GCS recorded
3 = 4/303 (1%) participants had missing data on IV dexamethasone administration
4 = 2/172 (1%) participants with confirmed S. pneumoniae meningitis had missing data on IV dexamethasone administration
5 = 1/303 (0.3%) participants had missing data on ITU admission

On both univariate and multivariate analyses (adjusted for age and sex), in patients with confirmed bacterial meningitis, the administration of dexamethasone was associated with a reduction in in-hospital mortality (aOR 0.57, 95% CI 0.28 – 1.17, p 0.12). When this analysis was restricted to include only those with confirmed *Streptococcus pneumoniae* meningitis, those who received dexamethasone had a reduced odds of in-hospital mortality (aOR 0.47, 95% CI 0.20 – 1.10, p 0.08). Neither association reached statistical significance. This analysis

was also performed including the patients assumed to have bacterial meningitis according to the Spanos criteria (supplementary table 3).

Discussion

This large national study evaluated clinical management of adults with community acquired meningitis throughout the UK and Ireland. Current practice falls short of the recommendations in the 2016 UK guidelines(6). This is a concern for all patients but is of a particular worry in bacterial meningitis. The management of bacterial meningitis is time critical(4, 16). Delays in receiving antibiotics and having an LP, the unnecessary use of brain imaging, a lack of appropriate antibiotics in those at risk of *Listeria* and the low rate of steroid administration are areas for significant improvement.

Most patients were given antibiotics prior to LP. Even taking this into consideration, the median door to antibiotic time was over three hours. The optimal timing of antibiotics in bacterial meningitis is not known precisely but we do know that delays lead to increased mortality (4, 5, 16). A delay of over three hours has been associated with a 14-fold increase risk of death(16).

Delays in obtaining CSF are associated with a reduction in pathogen detection, increased exposure to unnecessary anti-infectives, prolonged hospital stays and increased mortality (4, 6, 17). In most cases brain imaging is not indicated in adults with suspected community-acquired meningitis (4) however, in our cohort, a significant number of patients had unnecessary scans. Although complications following LP are rare(18, 19), there may be an unfounded fear of cerebral herniation following LP, even in those with no clinical features of brain shift, which is leading to excessive use of imaging(20). Education programs, along with quality improvement measures, are essential to reduce the potentially harmful overuse of neuroloimaging. Additionally, it is essential that we optimise care pathways to ensure that

For peer review only

282 clinicians have the time, space and equipment required to performed LPs in a timely and safe manner (3, 21).

CSF culture positivity rates decline substantially when LP is delayed (3, 17). PCR can detect bacterial DNA in CSF for several days after antibiotics have been administered. In the UK half of meningococcal disease is diagnosed on PCR alone(22). It is alarming that PCR was used, in our cohort, as a diagnostic modality in so few patients. Meningitis specific investigation order-sets using electronic ordering, and/or reflex laboratory testing to increase use of molecular diagnostics should be considered to reduce opportunities for missed microbiological diagnoses. There is the potential for increased use of rapid technologies that can be used on site with minimal technical skill required(23). Having rapid tests on site has been shown to reduce bed days with significant cost savings(24). Further research evaluating rapid diagnostic tests in other types of meningitis with clinically relevant outcomes is needed. We also need to increase the offer of HIV testing in patients with meningitis, as less than half the patients had a documented HIV test. Incident HIV diagnoses were made in our cohort amongst patients presenting with bacterial, viral, and unknown cause meningitis.

There is good evidence that corticosteroids reduce mortality in pneumococcal meningitis with no clinically significant increase in adverse events in other causes of meningitis (25). Empirical steroids should be given for all adults with suspected bacterial meningitis. In our study, we saw a reduction in mortality in patients with pneumococcal meningitis who were given steroids, whilst this survival benefit did not reach statistical significance, this was likely due to a type two error and the small sample of confirmed pneumococcal meningitis cases. It is of concern that well-evidenced, well-established therapies known to improve outcome, including mortality, are only being given to just over half those who might benefit. A protocolised, goal-directed bundle, including the use of corticosteroids and appropriate antibiotics warrants evaluation in the UK. There were clear differences between centres in our

For peer review only

study with one centre administering steroids to 26/42 (63%) of their patients and another giving them to none. It is possible that those centres that adhered to the recommendation to give steroids may also have adhered to other aspects of the guidelines more often as well, contributing to improved outcomes.

Although this is a large multi-national study, there are limitations. NHS trusts self-selected themselves for inclusion, we cannot rule out any significant differences with trusts that did not. However, 64 hospitals were included with good representation throughout the nations of the UK (and Ireland). We don't think any potential selection bias limits the generalisability of our findings. We used well-established, published case definitions of meningitis to minimise information bias, however misclassification of cases remains possible especially in the cases without a confirmed microbiological diagnosis. Our case definitions allowed us to include anyone suspected of having meningitis (of any cause) as objectively it is often difficult to differentiate between viral and bacterial meningitis at the point of initial assessment. However, it is possible that there may have been differences in presentation between those with confirmed bacterial meningitis, those with confirmed viral meningitis and those with no confirmed aetiology that meant they were managed in different ways. This study was not powered to look at the differences between all the different aetiologies. Finally, because this was a retrospective study, our analysis may have been subject to errors resulting from recall bias and missing data. A prospective national study would have been challenging to execute and it is likely that there would have been ascertainment bias in time and geography. We therefore believe that, due to the large sample size along with the use of electronic hospital coding and laboratory data to ascertain cases, the risk of recall bias is low, and our retrospective data is representative of practice within the UK.

There is a clear need to better understand the sub-optimal guideline adherence reported here. Although there has been research regarding primary care practice there has not been any

evaluation of exactly where delays occur and what the barriers are to achieving good practice in secondary care(26, 27). A small questionnaire based study identified the inability to find correct equipment, lack of time and/or paucity of appropriately trained staff as potential barriers to performing timely lumbar puncture for the investigation of neurological infections(21).

Non-meningitis specific research evaluating barriers and facilitators to adhering to clinical guidelines, report a lack of awareness or familiarity with the guidelines, as well as disagreement with the content may both be important(28). External barriers such as equipment and staffing were also identified which agrees with the limited research that there is in neurological infections. There is observational evidence from other countries of improvements in practice and outcome following implementation of guidelines (12, 29).

The patient journey in the UK normally starts with being admitted via an emergency department or acute medical unit where clinicians may not be as familiar with the guidelines and evidence as specialists. There is some evidence, both within meningitis and other infectious diseases that management is improved by being looked after by a specialist. There is an expert recommendation within the current UK guidelines that patients with meningitis should be looked after with input of an infection specialist.

In conclusion this is, to our knowledge, the largest UK study of adult patients with meningitis. Awareness of practice guidelines for relatively rare acute medical conditions such as meningitis is low and this study has demonstrated that despite clear, freely accessible guidelines, clinical care is not in line with evidence-based recommendations. There is considerable room for improvement. Whilst we recognise that guidelines do not improve practice on their own, we do recommend that the findings from this study are strongly considered in the development of the new National Institute for Clinical Excellence (NICE) guideline on meningitis currently being developed, which for the first time, will include guidance for adult patients as well as children. Given the widespread adoption of NICE

For peer review only

endorsed guidelines and quality standards to improve the quality of clinical practice in the UK, we anticipate that a NICE guideline will improve awareness and uptake of good practice in the short term. In addition to education, which has limited impact on changing behaviour, UK hospitals should use quality improvement methods to improve management of patients with suspected meningitis. Good qualitative research to identify what the barriers to implementing the guidelines should also be done.

We suggest a national strategic improvement plan should focus on the following key areas: timely use of diagnostics; appropriate antibiotics in at risk populations and the use of adjunctive steroids. The integrated use of electronic systems to standardize optimal use of diagnostics, and management bundles may offer additional opportunities to improve outcomes. Each site that has been involved in this study has been asked to implement site specific changes and re-evaluate for any improvements in practice.

For peer review only

Contributors

Ellis J: Methodology, Data collection and curation, formal analysis, Investigation, Writing – Original Draft Preparation. Harvey D: Methodology including pilot data, data collection, reviewing and approving final draft. Defres S: Methodology including development of original audit tool and guidelines, data collection, reviewing and approving final draft. Chandna A: Methodology, reviewing and approving final draft. Maclachlan E: Methodology, data collection, reviewing and approving final draft. Solomon T: Methodology including development of original guidelines and audit tool, reviewing and approving final draft. Heyderman RS: Conceptualization, Methodology, Supervision, Writing – Review & Editing. McGill F: Conceptualization, Methodology, Data collection and curation, Investigation, formal analysis, Writing –Original Draft Preparation. Responsible for overall content as guarantor. The corresponding author attests that all listed authors meet authorship criteria and that no others meeting the criteria have been omitted. The lead author and corresponding author affirm that the manuscript is an honest, accurate, and transparent account of the study; no important aspects of the study have been omitted.

The appendix includes a list of other contributors in the National Audit of Meningitis Management (NAMM) group.

The Corresponding Author has the right to grant on behalf of all authors and does grant on behalf of all authors, a worldwide licence to the Publishers and its licensees in perpetuity, in all forms, formats and media (whether known now or created in the future), to i) publish, reproduce, distribute, display and store the Contribution, ii) translate the Contribution into other languages, create adaptations, reprints, include within collections and create summaries, extracts and/or, abstracts of the Contribution, iii) create any other derivative work(s) based on the Contribution, iv) to exploit all subsidiary rights in the Contribution, v) the inclusion of electronic links from the Contribution to third party material where-ever it may be located; and, vi) licence any third party to do any or all of the above.

Competing interests

All authors have completed the ICMJE uniform disclosure form at www.icmje.org/coi_disclosure.pdf and declare: no support from any organisation for the submitted work; no financial relationships with any organisations that might have an interest in the submitted work in the previous three years; no other relationships or activities that could appear to have influenced the submitted work.

For peer review only

Acknowledgments

TS is supported by the National Institute for Health Research (NIHR) Health Protection Research Unit in Emerging and Zoonotic Infections (Grant No. NIHR200907), NIHR Global Health Research Group on Brain Infections (No. 17/63/110), and the UK Medical Research Council's Global Effort on COVID-19 Programme (MR/V033441/1)

Funding

This research received no specific grant from any funding agency in the public, commercial or not-for-profit sector.

Data availability statement

Data can be made available to other researchers on reasonable request to the authors.

For peer review only

416

For peer review only

References

1. van de Beek D, de Gans J, Spanjaard L, Weisfelt M, Reitsma JB, Vermeulen M. Clinical Features and Prognostic Factors in Adults with Bacterial Meningitis. N Engl J Med. 2004;351:1849-59.

2. Okike IO, Ribeiro S, Ramsay M, Heath PT, Sharland M, Ladhani SN. Trends in bacterial, mycobacterial and fungal meningitis in England and Wales 2004-11: an observational study. Lancet Infect Dis. 2014;14:301-7.

3. McGill F, Griffiths MJ, Bonnett LJ, Geretti AM, Michael BD, Beeching NJ, et al. Incidence, aetiology, and sequelae of viral meningitis in UK adults: a multicentre prospective observational cohort study. Lancet Infect Dis. 2018;18(9):992-1003.

4. Proulx N, Frechette D, Toye B, Chan J, Kravcik S. Delays in the administration of antibiotics are associated with mortality from acute bacterial meningitis. QJM. 2005;98:291-8.

5. Bodilsen J, Dalager-Pedersen M, Schonheyder HC, Nielsen H. Time to antibiotic therapy and outcome in bacterial meningitis: a Danish population-based cohort study. BMC Infect Dis. 2016;16(392):1-7.

6. McGill F, Heyderman RS, Michael BD, Defres S, Beeching NJ, Borrow R, et al. The UK joint specialist societies guideline on the diagnosis and management of acute meningitis and meningococcal sepsis in immunocompetent adults. J Infect. 2016;72(4):405-38.

7. Stockdale AJ, Weekes MP, Aliyu SH. An audit of acute bacterial meningitis in a large teaching hospital 2005-10. QJM. 2011;104(12):1055-63.

8. Cullen MM. An audit of the investigation and initial management of adults presenting with possible bacterial meningitis. J Infect. 2005;50:120-4.

9. Brouwer M, van de Beek D. Viral meningitis in the UK: time to speed up. Lancet Infectious Diseases. 2018;18(9):930-1.

10. Kadambari S, Okike I, Ribeiro S, Ramsay ME, Heath PT, Sharland M, et al. Seven-fold increase in viral meningo-encephalitis reports in England and Wales during 2004-2013. J Infect. 2014;69(4):326-32.

11. McGill F, Tokarz R, Thomson EC, Filipe A, Sameroff S, Jain K, et al. Viral capture sequencing detects unexpected viruses in the cerebrospinal fluid of adults with meningitis. Journal of Infection. 2022;84(4):499-510.

12. Glimåker M, Johansson B, Grindborg Ö, Bottai M, Lindquist L, Sjölin J. Adult Bacterial Meningitis: Earlier Treatment and Improved Outcome Following Guideline Revision Promoting Prompt Lumbar Puncture. Clin Infect Dis. 2015;60(8):1162-9.

13. Public Health England. UK Standards for Microbiology Investigations. Investigation of Cerebrospinal fluid. 2017.

14. The Kings Fund. NHS hospital bed numbers: past, present, future 2021 [Available from: https://www.kingsfund.org.uk/publications/nhs-hospital-bed-numbers.

15. Spanos A, Harrell FE, Durack DT. Differential Diagnosis of Acute Meningitis. An Analysis of the Predictive Value of Initial Observations. JAMA. 1989;262(19):2700-7.

16. Auburtin M, Wolff M, Charpentier J, Varon E, Tulzo YL, Girault C, et al. Detrimental role of delayed antibiotic administration and penicillin-nonsusceptible strains in adult intensive care unit patients with pneumococcal meningitis: The PNEUMOREA prospective multicenter study. Crit Care Medicine. 2006;34:2758-65.

17. Michael B, Menezes BF, Cunniffe J, Miller A, Kneen R, Francis G, et al. Effect of delayed lumbar punctures on the diagnosis of acute bacterial meningitis in adults. Emerg Med J. 2010;27(6):433-8.

18. Costerus JM, Brouwer MC, Sprengers MES, Roosendaal SD, van der Ende A, van de Beek D. Cranial Computed Tomography, Lumbar Puncture, and Clinical Deterioration in Bacterial Meningitis: A Nationwide Cohort Study. . Clin Infect Dis. 2018;67(6):920-6.

19. Costerus JM, Brouwer MC, van de Beek D. Technological advances and changing indications for lumbar puncture in neurological disorders. Lancet Neurol 2018;17(3):268-78.

For peer review only

20. Glimaker M, Johansson B, Bell M, Ericsson M, Blackberg J, Brink M, et al. Early Lumbar Puncture in adult bacterial meningitis - rationale for revised guidelines. Scand J Infect Dis. 2013;45:657-63.
21. Defres S, Mayer J, Backman R, kneen R. Performing lumbar punctures for suspected CNS infections: experience and practice of trainee doctors. Br J Hosp Med. 2015;76(11):658-62.
22. Heinsbroek E, Ladhani SN, Gray S, Guiver M, Kaczmarski E, Borrow R, et al. Added value of PCR testing for confirmation of invasive meningococcal disease in England. J Infect. 2013;67:385-90.
23. Bouzid D, Zanella MC, Kerneis S, Visseaux B, May L, Schrenzel J, et al. Rapid diagnostic tests for infectious diseases in the emergency department. Clinical microbiology and infection : the official publication of the European Society of Clinical Microbiology and Infectious Diseases. 2020.
24. Giuleri SG, Chapuis-Taillard C, Manuel O, Hugli O, Pinget C, Wasserfallen JB, et al. Rapid detection of enterovirus in cerebrospinal fluid by a fully automated PCR assay is associated with improved management of aseptic meningitis in adult patients. J Clin Virol. 2015;62:58-62.
25. de Gans J, van de Beek D, European Dexamethasone in Adulthood Bacterial Meningitis Study Investigators. Dexamethasone in Adults with Bacterial Meningitis. N Engl J Med. 2002;347(20):1549-56.
26. Brennan CA, Somerset M, Granier SK, Fahey TP, Heyderman RS. Management of diagnostic uncertainty in children with possible meningitis: a qualitative study. Br J Gen Pract. 2003;53:626-31.
27. Granier S, Owen P, Pill R, Jacobson L. Recognising meningococcal disease in primry care: qualitative study of how general practitioners process clinical and contextual ifnormation. BMJ. 1998;316:276-9.
28. Barth JH, Misra S, Moberg Aakre K, Langlois MR, Watine J, Twomey PJ, et al. Why are clinical practice guidelines not followed? Clinical chemistry and laboratory medicine. 2016;54(7):1133-39.
29. Costerus JM, Brouwer MC, Bijlsma MW, Tanck MW, van der Ende A, van de Beek D. Impact of an evidence-based guideline on the management of community-acquired bacterial meningitis: a prospective cohort study. Clinical microbiology and infection : the official publication of the European Society of Clinical Microbiology and Infectious Diseases. 2016;22(11):928-33.

For peer review only

Supplementary table 1 . Risk factors for Listeria stratified by aetiology.

	Total cohort N (%)	Bacterial meningitis N (%)	Viral meningitis N (%)	Other meningitis† N (%)	P value
**N**	1,471 (100)	302 (21)	615 (42)	553 (38)	-
**Age >60 years**	235 (16)	126 (42)	27 (4)	79 (14)	<0.001
**Number immunocompromised by disease/medication***	60 (4)	14 (5)	18 (3)	28 (5)	0.23
**Number with Diabetes mellitus**	64 (4)	30 (10)	11 (2)	22 (4)	<0.001
**Number with a history of alcohol excess**	36 (2)	21 (7)	3 (0.5)	12 (2)	<0.001

†= other meningitis category included all patients without a confirmed bacterial or viral pathogen

*=Conditions listed as immunocompromising conditions included haematological malignancy (n=8), Other malignancy (n=8), solid organ transplant (n=6), liver cirrhosis (n=1), HIV (n=9), Pregnancy (n=2). Medicaion listed included Steroids (n=7), tocilizumab, ecolizumab and infliximab (n=6), Methotrexate (n=8), Mycophenolate (n=2), Azathioprine (n=3), 'chemotherapy' (n=4). (some patients had more than one immunocompromising condition/medication).

For peer review only

Supplementary table 2: Multivariate analysis of the association between baseline co-variates and in-hospital mortality in 266 patients with bacterial meningitis confirmed by CSF analysis using logistic regression modelling:

Baseline co-variate	N	In-hospital mortality N (%)[1]	Crude OR for in-hospital mortality (95% CI)	P-value	Adjusted OR for in-hospital mortality (95% CI)*	P-value†
**Sex**						
Male	147	15 (10.2)	1			
Female	118	7 (5.93)	0.55 (0.22-1.42)	0.21		
**Age group**						
≤ 18 years	16	0 (0)	-			
19 – 59 years	136	10 (7.35)	1			
≥ 60 years	113	12 (10.6)	1.50 (0.62-3.61)	0.37		
**Blood culture positive**						
No	130	8 (6.15)	1		1	
Yes	135	14 (10.4)	1.76 (0.71-4.38)	0.21	1.46 (0.58-3.71)	0.42
**GCS ≤ 13[2]**						
No	106	3 (2.83)	1		1	
Yes	132	17 (12.9)	5.05 (1.41-18.2)	0.006	4.41 (1.24-15.7)	0.009
**IV dexamethasone given[3]**						
No	124	10 (8.06)	1		1	
Yes	137	11 (8.03)	0.99 (0.41-2.43)	0.99	1.02 (0.41-2.52)	0.96
**IV dexamethasone given if *Strep.pneumoniae*[4]**						
No	62	8 (12.9)	1		1	
Yes	89	8 (8.99)	0.67 (0.23-1.89)	0.44	0.68 (0.24-1.94)	0.48
**Final diagnosis *S. pneumoniae***						
No	107	5 (4.46)	1		1	
Yes	136	17 (11.1)	2.67 (0.95-7.55)	0.05	2.37 (0.84-6.67)	0.08
**ITU admission[5]**						
No	129	4 (3.01)	1		1	
Yes	113	18 (13.7)	5.14 (1.65-16.0)	0.002	4.44 (1.44-13.6)	0.003

*adjusted for sex and age group
† P-value from LRT comparing models with and without primary exposure variable
1 = One participant had missing outcome data
2 = 28/266 (10%) participants did not have a GCS recorded
3 = 4/266 (1%) participants had missing data on IV dexamethasone administration
4 = 2/154 (1%) participants with confirmed S. pneumoniae meningitis had missing data on IV dexamethasone administration
5 = 2/266 (0.7%) participants had missing data on ITU admission

Supplementary table 3: Multivariate analysis of the association between baseline co-variates and in-hospital mortality in 359 patients with bacterial meningitis using the Spanos criteria^ using logistic regression modelling:

Baseline co-variate	N	In-hospital mortality N (%)[1]	Crude OR for in-hospital mortality (95% CI)	P-value	Adjusted OR for in-hospital mortality (95% CI)*	P-value†
**Sex**						
Male	199	28 (14.1)	1			
Female	159	13 (8.18)	0.54 (0.27-1.09)	0.08		
**Age group**						
≤ 18 years	21	0 (0)	-			
19 – 59 years	192	18 (9.38)	1			
≥ 60 years	145	23 (15.9)	1.82 (0.94-3.52)	0.07		
**Blood culture positive**						
No	188	14 (7.45)	1		1	
Yes	170	27 (15.9)	2.35 (1.18-4.68)	0.01	1.93 (0.96-3.89)	0.06
**GCS ≤ 13[2]**						
No	163	9 (5.52)	1		1	
Yes	156	28 (17.9)	3.74 (1.67-8.36)	<0.001	3.19 (1.44-7.09)	0.003
**IV dexamethasone given[3]**						
No	189	26 (13.8)	1		1	
Yes	162	14 (8.64)	0.59 (0.30-1.18)	0.13	0.57 (0.28-1.14)	0.11
**IV dexamethasone given if *Strep.pneumoniae*[4]**						
No	73	16 (21.9)	1		1	
Yes	96	11 (11.5)	0.46 (0.19-1.08)	0.07	0.47 (0.20-1.10)	0.08
**Final diagnosis *S. pneumoniae***						
No	187	13 (6.95)	1		1	
Yes	171	28 (16.4)	2.62 (1.30-5.29)	0.005	2.29 (1.14-4.63)	0.02
**ITU admission[5]**						
No	192	9 (4.69)	1		1	
Yes	163	32 (19.6)	4.97 (2.24-11.0)	<0.001	4.43 (2.03-9.68)	<0.001

*adjusted for sex and age group
† P-value from LRT comparing models with and without primary exposure variable
1 = One participant had missing outcome data
2 = 40/359 (11%) participants did not have a GCS recorded
3 = 7/359 (2%) participants had missing data on IV dexamethasone administration
4 = 2/172 (1%) participants with confirmed S. pneumoniae meningitis had missing data on IV dexamethasone administration
5 = 4/359 (1%) participants had missing data on ITU admission
^ - Spanos criteria use various parameters to allow patients who have not had an aetiological agent to be assumed to be likely bacterial in nature.

Site of Data collection	Names and Grades (at time of data collection) of contributors	Number of patients' data contributed
Birmingham Heartlands Hospital, University Hospitals Birmingham NHS Foundation Trust	Amy Chue, SpR	60
	Ed Moran, Consultant	
	Karishma Gokani, CMT	
North Manchester General Hospital	Joseph Thompson, SpR	54
North Manchester General Hospital	Katherine Ajdukiewicz, Consultant	54
Oxford University Hospitals	Victoria Ward, SpR	50
Oxford University Hospitals	Lucinda Barrett, Consultant	50
Cheltenham General Hospital	Frances Edwards, CMT	47
Cheltenham General Hospital	Adam Usher, Consultant	47
Royal Alexandra Hospital, Paisley	Mairi McLeod, Consultant	45
Royal Alexandra Hospital, Paisley	Ramandeep Singh, medical student	45
Royal Alexandra Hospital, Paisley	Su su Htwe, SpR	45
Leicester Royal Infirmary, Leicester	Benedict Rogers, SpR	42
Leicester Royal Infirmary, Leicester	Grace Duane, Medical Student	42
Leicester Royal Infirmary, Leicester	Martin Wiselka, Consultant	42
Leicester Royal Infirmary, Leicester	Nicholas Wong, SpR	42
NHS Lothian	Elen Vink, SpR	42
NHS Lothian	Jennifer Poyner, SpR	42
NHS Lothian	Jenni Crane, Consultant	42
NHS Lothian	Ollie Lloyd, SpR	42
NHS Lothian	Emma Chisholm, SpR	42
Countess of Chester Hospital	Ildiko Kustos, Consultant	40
Countess of Chester Hospital	Ruth McEwen, Consultant	40
Countess of Chester Hospital	Sam Sutton, CMT	40
University Hospitals Plymouth Trust	Lewis Jones, Consultant	38
University Hospitals Plymouth Trust	Robert Tilley, Consultant	38
Addenbrookes hospital, Cambridge University Hospitals NHS Foundation Trust	M. Estee Torok, Honorary Consultant	37
Addenbrookes hospital, Cambridge University Hospitals NHS Foundation Trust	Isobel Ramsay, SpR	37
Hull University Teaching Hospitals NHS Trust	Monica Ivan, Consultant	36
Hull University Teaching Hospitals NHS Trust	Joshua York	36
Hull University Teaching Hospitals NHS Trust	Jennifer Ansett	36
Hull University Teaching Hospitals NHS Trust	Maithili Varadarajan	36
Hull University Teaching Hospitals NHS Trust	Celestine Eshiwe, SpR	36
London King's College	Amanda Fife, Consultant	36
London King's College	Stephanie Harris, SpR	36
London King's College	Ryan Jayesinghe, medical student	36
London King's College	Priya Sekhon	36
Aintree University Hospital, Liverpool	James Cruise, SpR	35
Aintree University Hospital, Liverpool	Susan Larkin, Consultant	35
Worcestershire Royal Hospital	Shivani Kanabar, Medical student	35
Worcestershire Royal Hospital	Ernest Mutengesa, Medical Student	35
Worcestershire Royal Hospital	Mirella Ling, Consultant	35
Worcestershire Royal Hospital	Christopher Green, Consultant	35
Bristol Royal Infirmary, University Hospitals Bristol NHS Foundation Trust	Martin Williams, Consultant	33

For peer review only

Bristol Royal Infirmary, University Hospitals Bristol NHS Foundation Trust	Matthew Stevens, CMT	33
Victoria hospital, Kirkcaldy	David Griffith, Consultant	32
Victoria hospital, Kirkcaldy	Naomi Bulteel, SpR	32
Northumbria Healthcare NHS Foundatio Trust	Charlotte Milne, SpR	30
Northumbria Healthcare NHS Foundatio Trust	Jayanta Sarma, Consultant	30
Ninewells hospital, Dundee	Aline Wilson, SpR	29
Ninewells hospital, Dundee	John Shone, Consultant	29
Ninewells hospital, Dundee	Lynn Urquhart, Consultant	29
Ninewells hospital, Dundee	Sahar Eldirdiri, SpR	29
Royal Preston Hospital, Lancashire Teaching Hospitals NHS Foundation Trust	Alison Muir, Consultant	28
Royal Preston Hospital, Lancashire Teaching Hospitals NHS Foundation Trust	Leila White, Clinical Scientist	28
Sheffield teaching Hospitals	Jody Aberdein, Consultant	28
Sheffield teaching Hospitals	Phillip Simpson, SpR	28
Shrewbury and Telford Hospital NHS Trust	Hnin Hay Mar	26
Shrewbury and Telford Hospital NHS Trust	John Bowen	26
Shrewbury and Telford Hospital NHS Trust	Keying Tan	26
Shrewbury and Telford Hospital NHS Trust	Eint Shwe Zin thein	26
Shrewbury and Telford Hospital NHS Trust	Mahmoud Aziz	26
University Hospital North Midlands	Anthony Cadwgan, Consultant	25
University Hospital North Midlands	Brendan Davies, Consultant	25
University Hospital North Midlands	Daniel White, SpR	25
University Hospital North Midlands	Natasha Weston, SpR	25
University Hospital North Midlands	Salman Zeb, CMT	25
St George's Hospital, London	Angela Houston, Consultant	24
St George's Hospital, London	Imogen Fordham, clinical fellow	24
St George's Hospital, London	Terry John Evans, SpR	24
St George's Hospital, London	Louise Wootton, Physician's associate	24
Nottingham University Hospitals NHS Trust	David Turner, Consultant	24
Nottingham University Hospitals NHS Trust	Iona Willingham, SpR	24
Birmingham Queen Elizabeth Hospital	Aimee Johnson, SpR	23
Birmingham Queen Elizabeth Hospital	Nimal Wickramasinghe, Consultant	23
Salford Royal Infirmary, Salford	Ashley Horsley, SpR	23
Salford Royal Infirmary, Salford	Eamonn Trainor, Consultant	23
Salford Royal Infirmary, Salford	Olivier Gaillemin, Consultant	23
University Hospital Southampton NHS Foundation Trust	Andrew Rosser, Consultant	23
University Hospital Southampton NHS Foundation Trust	Nicholas J Norton, SpR	23
Royal Blackburn Hospital, East Lancashire Hospitals NHS Trust	Iain Crossingham, Consultant	22
Royal Blackburn Hospital, East Lancashire Hospitals NHS Trust	Katie Cheung, Medical Student	22
Royal Blackburn Hospital, East Lancashire Hospitals NHS Trust	Megan Duxbury, CMT	22
Queen Elizabeth University Hospital, NHS Greater Glasgow and Clyde	Ashutosh Deshpande, Consultant	22
Queen Elizabeth University Hospital, NHS Greater Glasgow and Clyde	Emilie Bellhouse, FY2	22
Queen Elizabeth University Hospital, NHS Greater Glasgow and Clyde	Kamaljit Khalsa, SpR	22
Imperial College School of Medicine	Helena Brezovjakova, Medical Student	22
Imperial College School of Medicine	Emma McLean, medical student	22

For peer review only

Imperial college healthcare NHS trust	Tanmay, Kanitkar, CMT	22
Imperial college healthcare NHS trust	Nicholas Davies, Consultant	22
Imperial College School of Medicine	Alexsander Dawidziuk, Medical Student	22
St James University hospital, Leeds	Eloisa Mclaughlin, Medical student	22
St James University hospital, Leeds	Joanna Allen, Consultant	22
St James University hospital, Leeds	Razan Saman, SpR	22
St James University hospital, Leeds	Sarah Kelly, SpR	22
Royal Liverpool University Hospital, Liverpool	Hugh Adler, SpR	22
Royal Liverpool University Hospital, Liverpool	Sylviane Defres, Consultant	22
Arrowe Park Hospital, Wirral	David Harvey, Consultant	21
Arrowe Park Hospital, Wirral	Elshadai Ejere, FY2	21
Queen's hospital, Romford	Aarti Shah, Consultant	21
Queen's hospital, Romford	Yiwen Soo, FY1	21
Raigmore Hospital, Inverness	Wendy Beadles, Consultant	21
Raigmore Hospital, Inverness	Heather Sturgeon, Medical student	21
Raigmore Hospital, Inverness	Brodie Cameron, Medical Student	21
James Cook University Hospital, Middlesbrough	Ben Tomlinson, SpR	20
James Cook University Hospital, Middlesbrough	David Chadwick, Consultant	20
University Hospital Monklands	Claire McGoldrick, Consultant	20
University Hospital Monklands	Katie McDowell, FY2	20
Cumberland infirmary, Carlisle	Alastair Miller, Consultant	19
Cumberland infirmary, Carlisle	Clive Graham, Consultant	19
Cumberland infirmary, Carlisle	Mpho Molosiwa, FY2	19
Newcastle Upon Tyne NHS Foundation Trust	Ewan Hunter, Consultant	19
Newcastle Upon Tyne NHS Foundation Trust	Ruth Owen, Medical Student	19
Newcastle Upon Tyne NHS Foundation Trust	Katherine Flack	19
Airedale hospital, Airedale	Adrian Kennedy, Consultant	18
Bradford Royal Infirmary, Bradford	Amy Robinson, Consultant	16
Bradford Royal Infirmary	Phoebe Cross, SpR	16
Bradford Royal Infirmary	Fay Perry	16
University Hospital Wales	Vithusha Inpadhas	16
Aberdeen Royal Infirmary	Ali Khan, SpR	15
Aberdeen Royal Infirmary	Sarathy Selvam, FY2	15
Aberdeen Royal Infirmary	Vhairi Bateman, Consultant	15
Aberdeen Royal Infirmary	Jeremy Wong, Medica Student	15
Lancaster Royal Infirmary	Henry Wu, FY2	15
Lancaster Royal Infirmary	Monika Pasztor, Consultant	15
Whittington Hospital, London	Trupti Patel, Consultant	14
Whittington Hospital, London	Ajanthiha Karunakaran, Medical Student	14
Russells Hall Hospital, Dudley	Basma Soliman, CT1	13
Russells Hall Hospital, Dudley	Hassan Paraiso, Consultant	13
Glasgow Royal Infirmary	Mairi McLeod, Consultant	13
Glasgow Royal Infirmary	Su su Htwe, SpR	13
Glasgow Royal Infirmary	Anna Smith	13
James Paget University Hospitals NHS Foundation Trust	Andrew Blanshard, CMT	12
James Paget University Hospitals NHS Foundation Trust	Harish Reddy, Consultant	12

For peer review only

Portsmouth Hospitals University NHS Trust	Avneet Shahi, SpR	12
Portsmouth Hospitals University NHS Trust	Helen Chesterfield, Consultant	12
Portsmouth Hospitals University NHS Trust	Oliver Bannister, CMT	12
Withybush hospital, Haverford West	Ben Schroeder, Medical Student	12
Withybush hospital, Haverford West	Ken Woodhouse, Consultant	12
Ashford and St Peter's NHS Foundation Trust	Jan Coebergh, Consultant	11
Ashford and St Peter's NHS Foundation Trust	Viva Levee, FY2	11
Mater Misericordiae University Hospital, Dublin	Eavan Muldoon, Consultant	11
Mater Misericordiae University Hospital, Dublin	Rhea O'regan, SPR	11
Mater Misericordiae University Hospital, Dublin	Tee Keat Teoh, SpR	11
Newham Hospital, Barts Health NHS Trust	Sathyavani Subbarao, SpR	11
Newham Hospital, Barts Health NHS Trust	Simon Tiberi, Consultant	11
Newham Hospital, Barts Health NHS Trust	Caryn Rosmarin	11
London UCL and Hospital for Tropical diseases at University College London Hospitals NHS Foundation Trust.	Jayne Ellis, SpR	10
London UCL and Hospital for Tropical diseases at University College London Hospitals NHS Foundation Trust.	Lucy Bell, CMT	10
London UCL and Hospital for Tropical diseases at University College London Hospitals NHS Foundation Trust.	Robert Heyderman, Consutant	10
Barts Health NHS Trust	Jonathan Lambourne, Consultant	10
Barts Health NHS Trust	Emma McGuire, SpR	10
Barts Health NHS Trust	Robert Serafino, Consultant	10
Guy's and St Thomas' NHS Foundation Trust	Anna Goodman, Consultant	9
Guy's and St Thomas' NHS Foundation Trust	Ishaan Bhide, FY1	
Guy's and St Thomas' NHS Foundation Trust	Karanjeet Sagoo, Medical Student	
Whipps Cross, Barte Health NHS Trust	Mark Melzer, Consultant	8
Whipps Cross, Barte Health NHS Trust	Maria Krutikov, SpR	8
The Royal Free Hospital, London	Indran Balakrishnan, Consultant	6
The Royal Free Hospital, London	Susan Hopkins, Consultant	6
The Royal Free Hospital, London	Tim Jones, SpR	6
Trafford General Hospital, Manchester University NHS Foundation Trust	Kajal Patel, Medical Student	4
Trafford General Hospital, Manchester University NHS Foundation Trust	Barzo Faris, Consultant	
William Harvey Hospital, East Kent	Graeme Calv er, Consultant	3
William Harvey Hospital, East Kent	Ricky Singh, Medical Student	3
William Harvey Hospital, East Kent	Hazel Sanghvi, Medical Student	3
Tameside General Hospital	Mohamed Eltayeb, Clinical Fellow	2
Tameside General Hospital	Rathur Haris, Consultant	2

For peer review only

STROBE Statement—Checklist of items that should be included in reports of ***cohort studies***

	Item No	Recommendation	Location in paper
**Title and abstract**	1	(*a*) Indicate the study's design with a commonly used term in the title or the abstract	Title, page 1, line 1
		(*b*) Provide in the abstract an informative and balanced summary of what was done and what was found	Abstract, page 2, line 40 onwards
**Introduction**			
Background/rationale	2	Explain the scientific background and rationale for the investigation being reported	Introduction, page 4 line 80 onwards
Objectives	3	State specific objectives, including any prespecified hypotheses	Introduction, page 5, line 104
**Methods**			
Study design	4	Present key elements of study design early in the paper	Methods, page 5, line 108 onwards
Setting	5	Describe the setting, locations, and relevant dates, including periods of recruitment, exposure, follow-up, and data collection	Methods, page 5, line 108 onwards
Participants	6	(*a*) Give the eligibility criteria, and the sources and methods of selection of participants. Describe methods of follow-up	Methods, page 6, box 1
		(*b*) For matched studies, give matching criteria and number of exposed and unexposed	NA
Variables	7	Clearly define all outcomes, exposures, predictors, potential confounders, and effect modifiers. Give diagnostic criteria, if applicable	Methods, page 6, box 1 and line 125 onwards
Data sources/ measurement	8*	For each variable of interest, give sources of data and details of methods of assessment (measurement). Describe comparability of assessment methods if there is more than one group	Methods, page 5, line 110 onwards
Bias	9	Describe any efforts to address potential sources of bias	Methods, page 5, line 117 onwards Discussion, page 17, line 294 onwards
Study size	10	Explain how the study size was arrived at	Methods, page 5, line 110 onwards
Quantitative variables	11	Explain how quantitative variables were handled in the analyses. If applicable, describe which groupings were chosen and why	Methods, page 7, line 141 onwards
Statistical methods	12	(*a*) Describe all statistical methods, including those used to control for confounding	Methods, page 7, line 141 onwards
		(*b*) Describe any methods used to examine subgroups and interactions	NA
		(*c*) Explain how missing data were addressed	Methods, page 6, line 130
		(*d*) If applicable, explain how loss to follow-up was addressed	NA
		(*e*) Describe any sensitivity analyses	NA

Results

For peer review only - http://bmjopen.bmj.com/site/about/guidelines.xhtml

Participants	13*	(a) Report numbers of individuals at each stage of study—eg numbers potentially eligible, examined for eligibility, confirmed eligible, included in the study, completing follow-up, and analysed	Results, page 7, line 156
		(b) Give reasons for non-participation at each stage	NA
		(c) Consider use of a flow diagram	NA
Descriptive data	14*	(a) Give characteristics of study participants (eg demographic, clinical, social) and information on exposures and potential confounders	Page 9, Table 1
		(b) Indicate number of participants with missing data for each variable of interest	Page 10, Table 2
		(c) Summarise follow-up time (eg, average and total amount)	NA
Outcome data	15*	Report numbers of outcome events or summary measures over time	Page 9 Table 1, page 10, table 2 and page 15, table 3
Main results	16	(*a*) Give unadjusted estimates and, if applicable, confounder-adjusted estimates and their precision (eg, 95% confidence interval). Make clear which confounders were adjusted for and why they were included	Results, page 14, line 230 onwards
		(*b*) Report category boundaries when continuous variables were categorized	NA
		(*c*) If relevant, consider translating estimates of relative risk into absolute risk for a meaningful time period	NA
Other analyses	17	Report other analyses done—eg analyses of subgroups and interactions, and sensitivity analyses	NA
**Discussion**			
Key results	18	Summarise key results with reference to study objectives	Discussion, page 16 line 249 onwards
Limitations	19	Discuss limitations of the study, taking into account sources of potential bias or imprecision. Discuss both direction and magnitude of any potential bias	Discussion, page 17, line 294 onwards
Interpretation	20	Give a cautious overall interpretation of results considering objectives, limitations, multiplicity of analyses, results from similar studies, and other relevant evidence	Conclusion, page 18, line 313 onwards
Generalisability	21	Discuss the generalisability (external validity) of the study results	Discussion, page 17 line 296-8
**Other information**			
Funding	22	Give the source of funding and the role of the funders for the present study and, if applicable, for the original study on which the present article is based	Page 22, Line 439

*Give information separately for exposed and unexposed groups.

Note: An Explanation and Elaboration article discusses each checklist item and gives methodological background and published examples of transparent reporting. The STROBE checklist is best used in conjunction with this article (freely available on the Web sites of PLoS Medicine at http://www.plosmedicine.org/, Annals of Internal Medicine at http://www.annals.org/, and Epidemiology at http://www.epidem.com/). Information on the STROBE Initiative is available at http://www.strobe-statement.org.

For peer review only

For peer review only - http://bmjopen.bmj.com/site/about/guidelines.xhtml

For peer review only

For peer review only - http://bmjopen.bmj.com/site/about/guidelines.xhtml
